# Hydrochemical and isotopic signatures of groundwater infiltration and legacy nitrogen discharge within Jeju Island aquaculture systems

**DOI:** 10.1038/s41598-025-26436-9

**Published:** 2026-01-06

**Authors:** Seung-Hee Kim, Sung-Eun Park, Young-Shin Go, Chung-Sook Kim, Changmin Kim, Minjune Yang, Taejin Kim, Dong-Hun Lee

**Affiliations:** 1 Institute of Sustainable Earth and Environmental Dynamics (SEED), Pukyong National University, 365 Sinseon-ro, Nam-gu, Busan, 48547 South Korea; 2 Marine Environment Research Division, National Institute of Fisheries Science, Busan, 46083 South Korea; 3 Environment and Fisheries Resource Research Division, West Sea Fisheries Research Institute, National Institute of Fisheries Science, Incheon, 22383 Republic of Korea; 4 Division of Earth and Environmental System Sciences, Pukyong National University, Busan, 48513 South Korea; 5 Wible Co Ltd, Pukyong National University, 365, Sinseon-ro, Nam-gu, Busan, 48547 South Korea; 6 Marine Environmental Impact Assessment Center, National Institute of Fisheries Science, Busan, 46083, South Korea

**Keywords:** Saline groundwater, Nitrate, Organic matter, Stable isotope, Bayesian mixing model, Biogeochemistry, Ecology, Ecology, Environmental sciences

## Abstract

The terrestrial aquaculture systems in Jeju Island have been increasingly threatened by the intrusion of deteriorated groundwater. In this study, we investigated spatiotemporal variations in hydrochemical properties and isotopic compositions (δ^13^C_POC_, δ^15^N_PN_, δ^15^N_NO3_, and δ^18^O_NO3_) of influent waters from land-based fish farms to trace the sources of particulate organic matter (POM) and nitrate (NO₃⁻) pollution. During heavy summer precipitation (> 350 mm), hydrochemical and isotopic shifts revealed increased infiltration of terrestrial groundwater into fish farm systems. Based on discriminative isotopic values of POM and NO₃⁻ in aquaculture influents, source apportionment results after accumulated precipitation indicated that the contribution of anthropogenic POM (livestock; 39 ± 17%) was predominant than those of other sources (soil; 31 ± 16%, fertilizer; 24 ± 7%, septic waste; 6 ± 1%). In addition, the contribution of NO₃⁻ sources (soil; 67 ± 7%, livestock; 18 ± 10%, septic waste; 9 ± 4%, fertilizer; 5 ± 1%) were largely controlled by specific land-use types adjacent to aquaculture systems. These results suggest that variations in volcanic bedrock permeability and land-use patterns may lead to distinct infiltration pathways and delayed responses between the northern and southern sections of Jeju Island. Therefore, these findings demonstrated that legacy pollutants stored in both the unsaturated and saturated zones may continue to impair groundwater quality, thereby affecting aquaculture water quality, particularly during periods of accumulated precipitation. To effectively manage nutrient-driven degradation in aquaculture systems, we recommend integrating isotopic source-specific monitoring with hydrogeological zoning and the targeted pretreatment of saline groundwater. This approach can mitigate eutrophication risks and enhance the sustainability of fish farming operations in volcanic islands.

**Supplementary Information:**

The online version contains supplementary material available at 10.1038/s41598-025-26436-9.

## Introduction

Over the past century, increasing anthropogenic activities have significantly elevated organic matter (OM) and nutrients loading in natural environments, resulting in adverse environmental consequences, such as eutrophication and soil acidification^1,2^. Among various nutrient species, nitrate (NO₃⁻) contamination in groundwater has emerged as a critical issue due to its persistence and mobility within aquifers^3–5^. The risk of groundwater NO₃⁻ contamination is primarily governed by two key factors: the intrinsic vulnerability of the aquifer and the magnitude of contamination induced by anthropogenic sources^6,7^. Intrinsic vulnerability is governed by hydrogeological characteristics which influence the transport and attenuation of contaminants, whereas the magnitude of external contamination is largely governed by land-use practices, agricultural runoff, industrial discharge, and wastewater infiltration^8–10^. Especially, the excessive usages of fertilizers and the burial of residential and livestock wastes in the subsurface can continuously discharge amounts of OM and NO_3_^−^ into the groundwater for decades^4,8^. Therefore, given the increasing global concerns for anthropogenic N contamination^11–13^, systematic tracking of these risk factors is crucial for developing effective mitigation strategies and sustainably manage groundwater resources.

Among the emerging concerns for anthropogenic N contamination within groundwater, the expansion of intensive land-based aquacultures (e.g., fish farms) has significantly influenced the surrounding environment, particularly the quality of shallow groundwater^14–16^. For the operation of aquaculture systems, a large volume of culture water is substantially supplied by ambient saline groundwater, resulting in the discharge of nutrient-rich effluents, aquaculture-derived OMs, and residual feed into adjacent soils and coastal regions^15–17^. Under these hydraulic connections between terrestrial and coastal areas, the continuous circulation of rearing water within land-based aquaculture leads to deteriorates groundwater quality, mainly due to the mixing of organic/inorganic N compounds (e.g., urea, NO_3_^−^, NH_4_^+^) and including pollutants (i.e., sewage, fertilizer, manure, waste water) within terrestrial-coastal systems^4,8,18^.In this context, the precise identification of anthropogenic-derived organic/inorganic N sources is important for effectively mitigating anthropogenic N contamination within saline groundwater. Actually, mass mortality events of cultured fish (i.e., bastard halibut *Paralichthys olivaceus*) have been repeatedly reported in Jeju Island^19,20^, highlighting the urgent need to address deteriorating groundwater quality and its impacts on aquaculture sustainability. However, due to the presence of the mixed pollution sources preserved within groundwater and the hydrological time lag of accumulated OM and NO₃⁻ levels^4,21–23^ the systematic classification of contaminated anthropogenic N sources remains a challenge. In this regard, the use of isotopic compositions of particulate OM (POM, δ¹^3^C and δ¹^5^N) and NO₃⁻ (δ¹⁵N and δ¹⁸O) has been widely recognized as a valuable tool for identifying mixed pollution sources in water, including anthropogenic (e.g., synthetic fertilizers and manure/septic wastes) and natural (e.g., soil, and C_3_ plant) sources^24–27^. In addition, NO₃⁻ isotopic compositions in water resources provide valuable insight into nitrogen transformation processes, such as assimilation, nitrification and denitrification, further enhancing the understanding of OM and nutrient interaction dynamics in hydrological systems^26–29^.

Jeju Island (with a total area of 1848 km^2^), the largest volcanic island located in the southern part of the Korean Peninsula, is mainly composed of porous basaltic rocks^4,8,29^. Because of their high permeability and low storage capacity, these volcanic rocks serve as the primary aquifer system of the island but are also highly vulnerable to contamination from surface pollutants, including livestock manure and synthetic fertilizers^29–32^. Furthermore, legacy organic waste from illegal disposal can interact with infiltrating precipitation, releasing organic and inorganic N species that subsequently percolate into the groundwater Tables^4,8^. Therefore, in such conditions, the effective management of increased N sources within groundwater is necessary for mitigating the effects of severe anthropogenic N contamination that has occurred over several decades^33–35^. In relation to the mass mortality events of cultured fish, it is noteworthy that the rearing water within these culture facilities is typically composed of a mixture of offshore seawater (sourced from approximately 500 m offshore) and groundwater (extracted from a depth up to 100 m) to maintain optimal conditions for rearing fish (temperature: 17^_^18°C and pH: approximately 7.2). Therefore, to better understand the linkage between persistent anthropogenic N contamination and the mortality of farmed fish, source tracing of the mixed N origins preserved within the influent of land-based aquacultures is required to systematically establish effective water quality management strategies.

To address this, we conducted a case study on land-based aquaculture systems, focusing on Jeju Island, where intensive aquaculture operations (around 360 fish farms) have recently expanded. Based on the hydrochemical properties of aquaculture influents, our main objectives were to (1) identify different origins of POM and NO_3_^−^ sources using stable isotopic compositions, and (2) determine the discriminative contribution between natural and anthropogenic sources. To this end, we established a systematic framework for effectively managing anthropogenic N sources transported into the influents of land-based aquaculture systems.

## Results

### Temporal variation of influent water hydrochemical properties

**Fig. 1:**
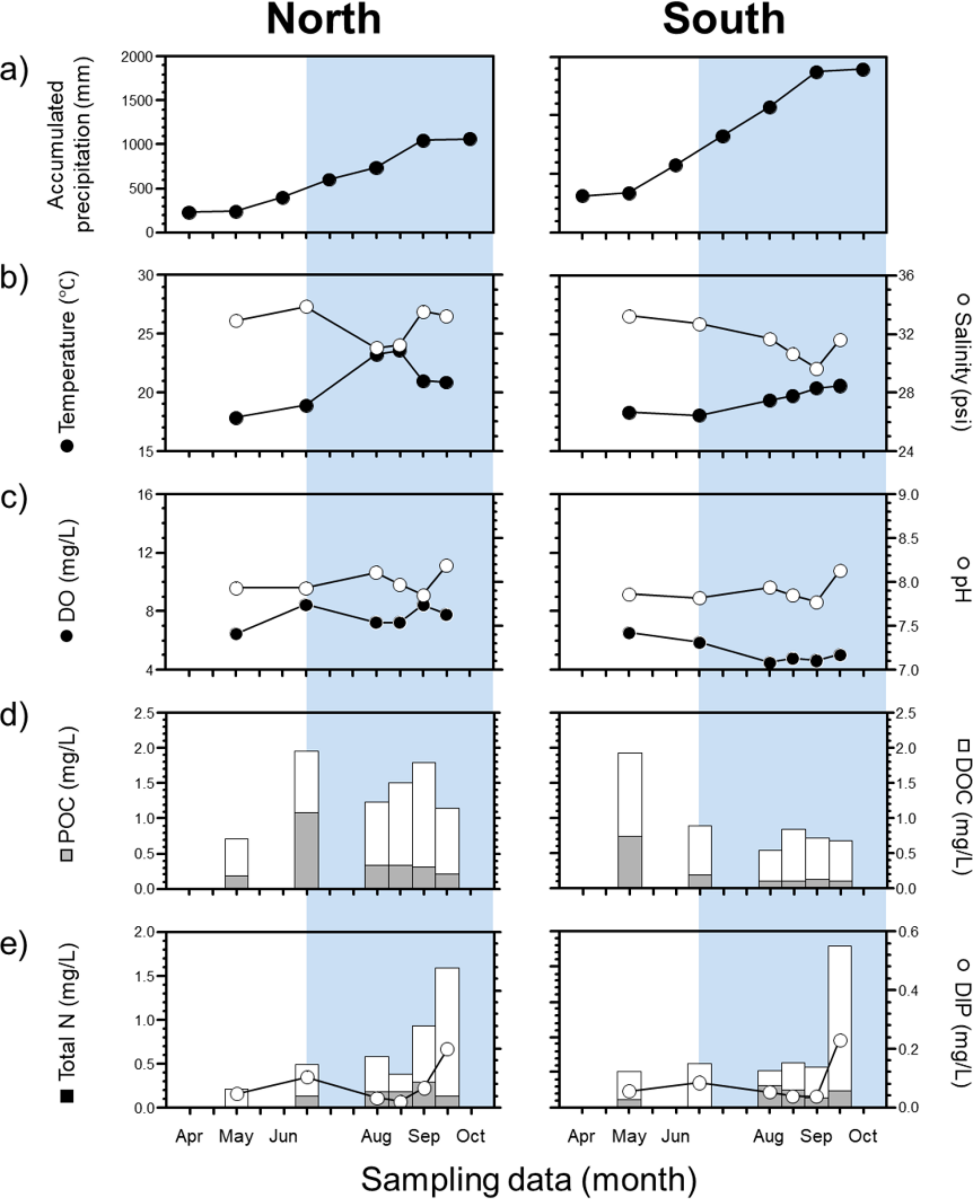
Temporal variations in (**a**) accumulated precipitation and physicochemical properties (**b**) temperature and salinity, (**c**) dissolved oxygen and pH, (**d**) organic carbon, and (**e**) total nitrogen and dissolved inorganic phosphate of fish farm influent water from northern and southern sections. Blue shaded areas indicate the post-precipitation period (July to September 2022).

The physicochemical properties of the fish farm influents showed discriminative spatiotemporal variations in response to increased summer precipitation (Fig. 1). Under the accumulated precipitations (17.8–1558.2 mm; Fig. 1a), the temperature and salinity varied from 20.5 ± 3.2 °C and 32.6 ± 1.4 PSU for the northern sections to 19.5 ± 1.3 °C and 32.0 ± 1.4 PSU for the southern sections, respectively (Fig. 1b). DO and pH values were 7.3 ± 1.3 mg/L and 8.0 ± 0.2 for northern sections; and 5.6 ± 1.0 mg/L and 7.9 ± 0.1 for southern sections, respectively (Fig. 1c). The concentrations of particulates and dissolved organic/inorganic factions exhibited spatiotemporal variations in the aquaculture influents (Fig. 1d, e). The concentrations of POC and DOC were 0.3 ± 0.1 mg/L and 1.0 ± 0.6 mg/L for north sections, and 0.3 ± 0.4 mg/L and 0.9 ± 0.6 mg/L for south sections (Fig. 1d). The concentrations of PN and DIN ranged were 0.1 ± 0.1 mg/L and 0.5 ± 0.6 mg/L for north sections, and 0.2 ± 0.1 mg/L and 0.6 ± 0.6 mg/L for south sections (Fig. 1e). DIP and DSi concentrations were found to be 0.1 ± 0.1 mg/L and 4.4 ± 5.6 mg/L for north sections, and 0.1 ± 0.1 mg/L and 3.9 ± 4.7 mg/L for south sections (Fig. 1e and Supplementary Table S1

### Isotopic composition of particulate organic matter (POM) and nitrate

**Fig. 2:**
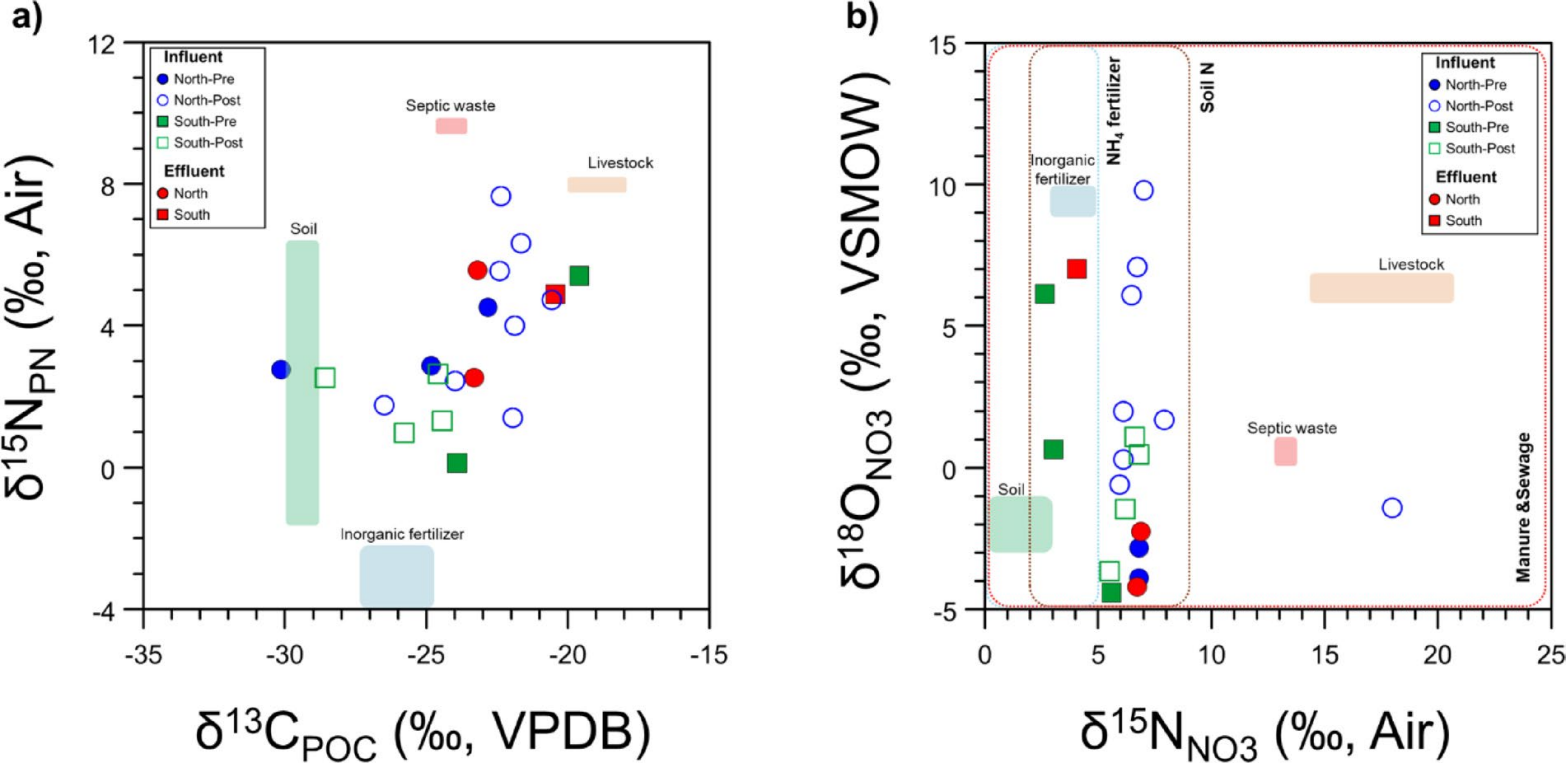
Spatiotemporal variations in isotopic compositions of (**a**) POM and (**b**) nitrate within fish farm influents and effluents from northern and southern sections.

The δ^13^C_POC_ and δ^15^N_PN_ values ranged from − 23.6 ± 2.6‰ and 3.2 ± 3.2‰ for northern sections to -24.3 ± 3.1‰ and 2.6 ± 2.0‰ for southern sections (Fig. 2a). For nitrate, the δ^15^N_NO3_ and δ^18^O_NO3_ values ranged from 7.6 ± 3.3‰ and 1.0 ± 4.5‰ for northern sections to 5.0 ± 1.6‰ and 0.7 ± 4.1‰ for southern sections, respectively (Fig. 2b). Regional source end-members collected within Jeju Island represented source specific isotopic signatures ranges for POM (soil: -29.4 ± 0.7‰ for δ^13^C_POC_ and 2.4 ± 4.6‰ for δ^15^N_PN_; septic waste: -24.1 ± 0.6‰ for δ^13^C_POC_ and 9.6 ± 0.3‰ δ^15^N_PN_; livestock: -19.0 ± 1.2‰ for δ^13^C_POC_ and 8.0 ± 0.2‰ for δ^15^N_PN_; inorganic fertilizer: -26.0 ± 1.5‰ for δ^13^C_POC_ and − 3.2 ± 1.2‰ for δ^15^N_PN_) and NO_3_ (inorganic fertilizer: 3.9 ± 1.2‰ for δ^15^N_NO3_ and 9.4 ± 0.6‰ for δ^18^O_NO3_; livestock: 17.6 ± 3.7‰ for δ^15^N_NO3_ and 6.4 ± 0.6‰ for δ^18^O_NO3_; soil: 1.5 ± 1.7‰ for δ^15^N_NO3_ and − 2.0 ± 1.2‰ for δ^18^O_NO3_; septic waste: 13.3 ± 0.6‰ for δ^15^N_NO3_ and 0.6 ± 0.6‰ for δ^18^O_NO3_), respectively (Supplementary information Table S4).

### Relative source contribution of POM and nitrate

**Fig. 3:**
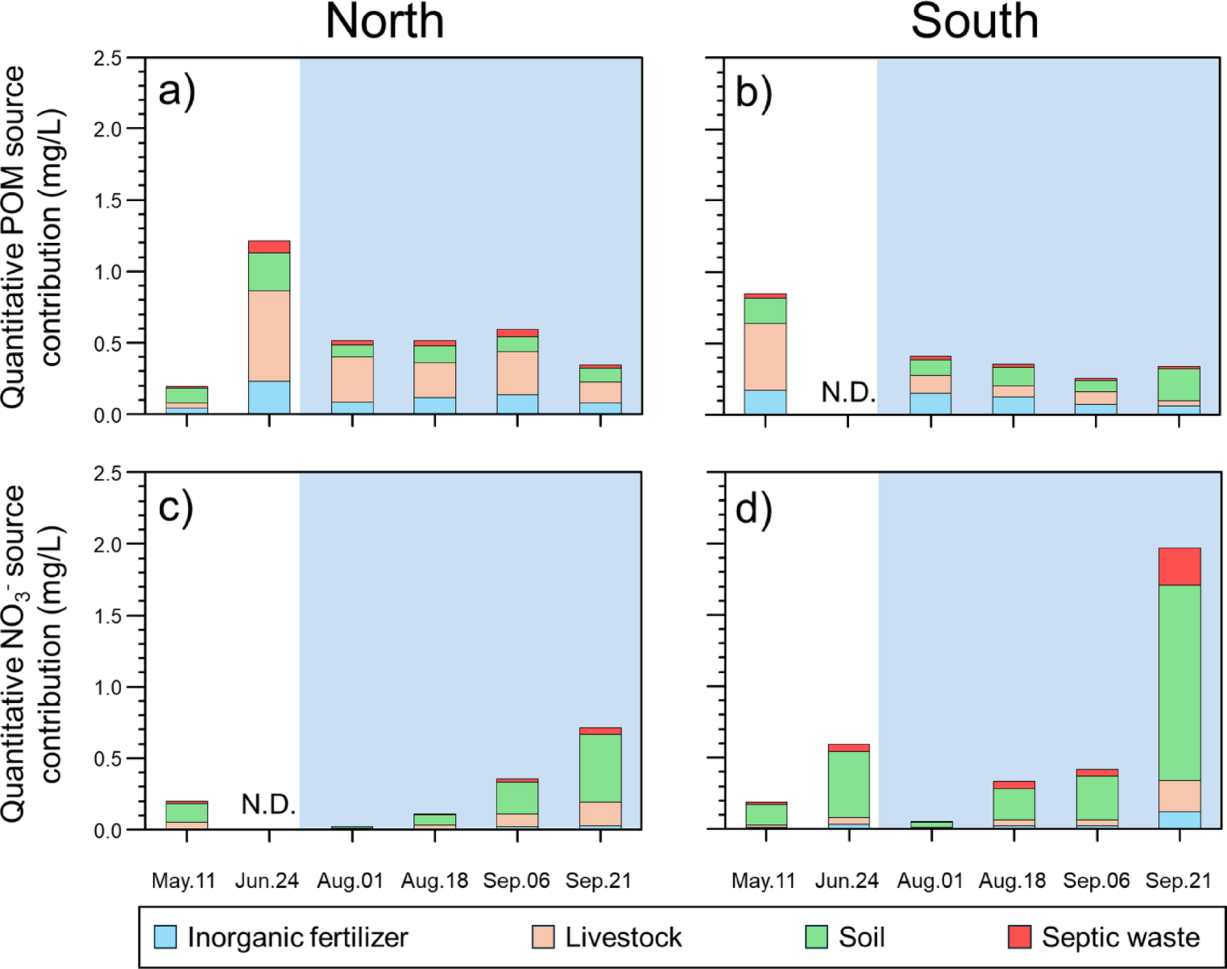
Spatiotemporal variations in quantitative source appointments of POM (**a**, **b**) and NO_3_^−^ (**c**, **d**) within fish farming influents from northern and southern sections. Each source fraction is represented by distinct colors (blue: inorganic fertilizer, peach: livestock, green: soil, red: septic waste). N.D. indicates “not determined”. Blue shaded areas indicate the post-precipitation period (July to September 2022).

The Bayesian model approach (i.e., MixSIAR) based on the isotopic compositions of POM and NO_3_ was applied to estimate the source contribution of anthropogenic origins intruding into fish farm influents (Fig. 3 and Supplementary Fig. S5). For POM fractions, the relative contribution of livestock (19–62%) was more predominant compared to other sources (inorganic fertilizer: 17–24%, soil: 16–54%, septic waste: 5–8%) in northern sections. Meanwhile, soil (21–67%) was the most dominant source compared to the others in southern sections, with inorganic fertilizer (19–37%), livestock (10–55%), and septic waste (4–7%) accounting for the remaining sources (Supplementary Fig. S5). For NO_3_ fractions, the relative contribution from soil N was the highest in both sections (northern: 51–67%; southern: 66–78%) compared to other sources (inorganic fertilizer: 3–5% for north and 6–7% for south; livestock: 23–39% for north and 8–12% for south; septic waste: 6–7% for north and 8–15% for south).

## Discussions

### Spatiotemporal variation in hydrochemical properties in fish farm influents

Generally, the hydrochemical properties of aquatic systems are strongly influenced by local precipitation, which directly affects the inflow to the water column, hydrological dilution of chemical components, surface runoff, groundwater infiltration, and the transport of chemical constituents into river and groundwater systems^9,36,37^. Considering that sustained water discharge via surface runoff into the local rivers and streams is largely negligible on Jeju Island^38,39^, the observed anomalies in physical properties of fish farm influents (temperature: 0.1–5.0 °C; salinity: 0.1–2.7 PSU; pH: approximately 0.3) are likely influenced primarily by the groundwater flow driven by accumulated precipitation^16,40^. Under such conditions, the discriminative hydrochemical responses between the northern and southern sections may be attributed to the differing time-delayed effects related to groundwater residence times^16,41^. Actually, the northern section showed a relatively short residence time (24–54 years), due to the geographical and topographical conditions near Mt. Halla as reported by previous literatures^4,29,42^. In particular, because of the contrasting bedrock formations between the two regions (north: quaternary basaltic groups and sand deposits; south: quaternary basaltic groups), active water–rock interactions in the northern sections may be influenced by more permeable compositions compared to those in the southern sections (Supplementary Fig. S1). In addition, the potential water-rock interactions within the groundwater system have often been estimated through the cation and anion compositions of water samples^43,44^. Overall, these properties measured in fish farm influents, groundwater wells, stream freshwater, and coastal seawater showed discriminative variations between Na-Cl and Ca-Cl types in the Piper diagram (Supplementary Fig. S2), indicating the representative characteristics of saltwater preserved within the fish farm influents^45,46^. Temporal variations in major ions (SO_4_^2−^, HCO_3_^−^, Na^+^, K^+^, Ca^2+^, and Mg^2+^) in the water resources showed significant positive correlations with chloride (Cl^−^) across all study sections (Supplementary Fig. S3), indicating typical mixing behavior at terrestrial-marine interface. In this respect, compared to the hydrogeological properties of terrestrial water resources (groundwater and freshwater), fish farm influents in the northern sections could have continuously interacted with the fluctuating sub-groundwater flows within the aquifer surrounding various land-use types^47,48^. These results suggest that aquifers in the northern region are more dynamically connected to recent precipitation events, whereas aquifers in the southern region exhibit delayed responses due to lower permeability. To quantitatively validate these time-lagged responses and identify the legacy nitrate storage and release dynamics, future studies should incorporate groundwater age-dating methods such as tritium-helium or sulfur hexafluoride (SF₆) tracers^4,8^. These approaches are thus essential for constraining the residence time of groundwater and understanding the historical accumulation of nitrate within saturated and unsaturated zones.

Under the discriminative hydrochemical conditions mentioned above, spatiotemporal variations in TOC (expressed as the sum of POC and DOC) can be used as a typical parameter that reflects the source contribution of various TOC origins, such as terrestrial plants, algae/bacteria, and other contaminants within aquatic carbon pools^49^. In this study, the variations between POC and DOC showed no significant correlation within fish farm influents in the northern sections (*r* = 0.2, *p* > 0.05), in contrast to those in the southern sections (*r* = 0.7, *p* < 0.05). Instead, temperature variations showed a positive correlation with DOC patterns (northern sections: *r* = 0.7, *p* < 0.01; southern sections: *r* = 0.7, *p* < 0.05; Supplementary Table S3). In addition, the contrast correlation between DOC and salinity within two sections (*r* = -0.4 for northern, *r* = 0.4 for southern) may reflect the hypothesis of discriminate freshwater-seawater balance and organic carbon intrusion during groundwater recharge events between two sections. Therefore, we suggest that hydrogeological components associated with groundwater discharge may influence TOC fluctuations in aquaculture influents. In this context, spatiotemporal variations in TOC within aquaculture influents may result from the inputs of various terrestrial organic carbon sources, permeating into the groundwater^15,16,50^. Together with the hydrogeological evidence for saline groundwater fluctuation, the degree of infiltration of these terrestrial-derived TOC in the northern sections appears to be increasing more than that in the southern sections. Meanwhile, the variations in the total N (TN) concentrations (expressed as the sum of PN and DIN) showed similar trends between the two sections (Fig. 1e). The elemental ratios (POC/PN) of POMs varied from 9.1 ± 10.1 for northern sections to 6.4 ± 7.5 for southern sections, indicating discriminatively decreased patterns under accumulated precipitations (Supplementary Table S1). With respect to the source identification of POM preserved within aquatic environments, their elemental ratios may likely be indicative of different signatures between natural and anthropogenic sources (terrestrial C_3_ and C_4_ plant: >12, phytoplankton: 5–11, anthropogenic source: 3–5)^51,52^. The decreasing pattern of POC/PN in the southern sections suggest an increased supply of anthropogenic particulate N sources within the fish farm influents (Supplementary Table S1). The increased DIN abundance (mainly NO_3_^−^) in fish farm influents in both sections may be closely linked to the increased infiltration of fresh groundwater during periods of cumulative rainfall^15,53^. In fact, DIN concentrations in the sub-groundwater of Jeju Island have partially exceeded the natural threshold (5.5 mg/L NO_3_^−^)^54^, leading to severe nitrate contamination of fresh groundwater. Considering that anthropogenic N sources can be easily infiltrated through the permeable volcanic structures of Jeju Island^4,8,29^, groundwater of varying ages preserved within aquifer may be vulnerable for potential N contamination as a legacy effect^4,8,55^. In this respect, we also estimated the nutrient status of fish farm influents to identify variations in elemental cycles under potential anthropogenic nutrient inflows.

**Fig. 4:**
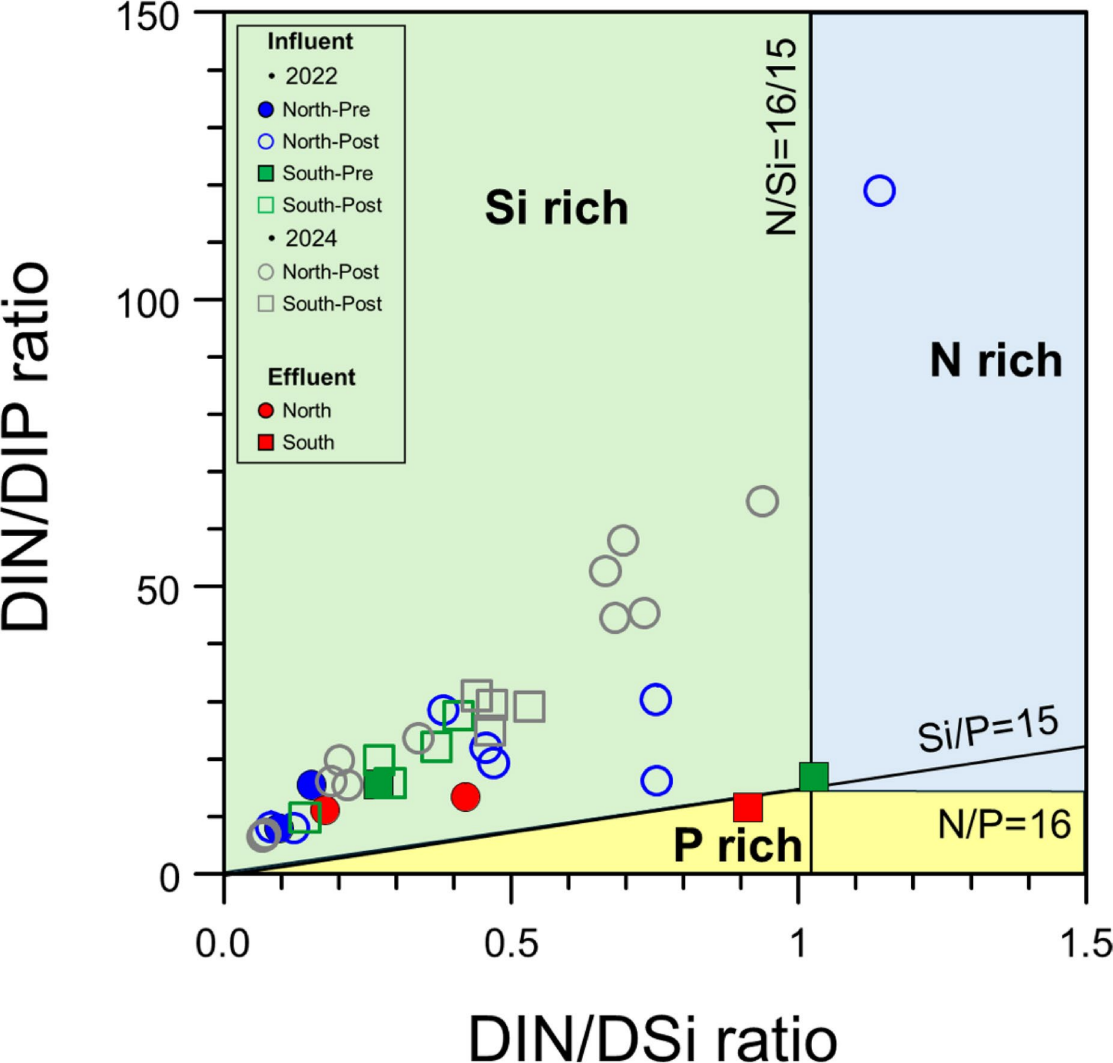
Temporal variations in nutrient status of fish farm influents from the northern and southern sections.

The nutrient status of the fish farm influents showed significantly different conditions during the period of increased precipitation (Fig. 4). Nutrient status (DIN/DIP and DIN/DSi) after precipitation events showed relatively N-rich conditions (DIN/DIP: 32.2 ± 35.8 for northern sections and 19.8 ± 7.4 for southern sections) compared to those of pre-precipitation periods (Supplementary Table S1). These N-rich conditions during accumulated precipitation may be regarded as a possible contribution of excess natural and anthropogenic N to the sub-groundwater system^11,37^. In this regard, the non-conservative characteristics of the hydrochemical components within the fish farm influent may reflect the substantial inflow of organic-inorganic sources as a result of the imbalance between fresh and saline groundwater (Supplementary Fig. S4). These relatively N-rich conditions and the non-conservative behavior observed during accumulated precipitation were also evident in the post-precipitation season of 2024, supporting the notion of excess terrestrial nutrient infiltration into the groundwater system under heavy rainfall events. The DIN variations within the fish farm influents were positively correlated with pH (northern sections: *r* = 0.6, *p* < 0.05; southern sections: *r* = 0.7, *p* < 0.01) and DIP (northern sections: *r* = 0.6, *p* < 0.05; southern sections: *r* = 0.7, *p* < 0.01; Supplementary Table S3). This correlation may suggest the increased infiltration of terrestrial nutrients during the accumulated precipitation^56–58^. Particularly, compared to N limitation (DIN/DIP: 12.0 ± 1.2) before precipitation (Fig. 4), the increased release of DIN and DIP with higher N/P ratios may potentially deteriorate water qualities within fish farm influents^16,59,60^. Under such conditions, an increased influx of inorganic nutrients (NO₃⁻, NH₄⁺, and PO₄³⁻) may cause physiologically negative effects on fish gills, internal organs, and hematological parameters, ultimately leading to the mortality of the aquaculture organisms^61–63^. Furthermore, the inorganic nutrients released though aquaculture effluent pipelines may directly contribute to the intensified blooms of green macroalgae (e.g., *Ulva* spp. including *U. conglobate* and *U. pertusa*) within the Jeju coastal ecosystem^15–17,53,64^. Therefore, to effectively manage the water quality of fish farm influent and effluents near Jeju coastal regions, it is essential to precisely identify the spatiotemporal variations in the sources of organic matter and inorganic nutrients. Thus, we estimated the source specificity by using the distinct isotopic signatures of particulate and dissolved constituents, thereby constraining the potential factors contributing to the variations in water quality within fish farm influents.

### Source tracing of POM and nitrate within fish farm influents

Overall, the isotopic signatures of POM exhibited regionally heterogeneous patterns, whereas their isotopic variations showed distinguishable differences between the pre- and post-precipitation conditions. Based on regional isotopic signatures collected within Jeju Island (Supplementary information Table S4.), isotopic variations of POM before precipitation may be inferred as the mixed origins of soil OM, inorganic fertilizer and livestock sources surrounded by different land-use types (i.e., agriculture, pasture, urban, forest; Supplementary Fig. S1, https://egis.me.go.kr/). Considering that groundwater infiltration may contain anthropogenic POM sources within fish farm influents, the overlapped isotopic signatures of POM may be regarded as mixed signatures of natural and anthropogenic sources under the prolonged residence time of the sub-groundwater. Meanwhile, after precipitation, δ^13^C_POC_ and δ^15^N_PN_ values ranged from − 22.7 ± 1.8‰ and 3.1 ± 3.9‰ for northern sections to -25.8 ± 1.9‰ and 1.9 ± 0.8‰ for southern sections (Fig. 2). These isotopic values showed a regionally significant difference (*p* < 0.05) with increasing POM concentration. Notably, according to the increased DIN/DIP ratio during accumulated precipitation, more ^15^N-enriched POM signatures in the northern sections may be involved in the increased infiltration of anthropogenic POM sources (e.g., septic waste and livestock) within the northern sub-groundwater. Alongside the highly permeable volcanic structure (e.g., basaltic groups and sand deposits) in the northern sections, these anthropogenic sources may have been rapidly transported from abundant quantities of POM within subsurface aquifer systems. In contrast, isotopic variations in the southern sections showed a greater predominance of ^15^N-depleted patterns under accumulated precipitation condition, indicating different origin sources (soil and inorganic fertilizer) within fish farm influents. The depletion of δ^15^N_PN_ values (1.0–2.7‰) likely reflects a predominant contribution from fertilizer and soil OM sources in the aquatic environments^65,66^. As the one of potential factors contributing to the deterioration of water quality of fish farm influents, the increased inflow of anthropogenic POM sources during accumulated precipitations can be gradually modulated through discriminative components (e.g., hydrogeological conditions and land-use types) between the northern and southern sections. Finally, with respect to increased concentration of N fractions (mainly ammonia) within Jeju coastal water^15,16^, the direct output of anthropogenic sources preserved within fish farm effluents may substantially influence the imbalance of elemental stoichiometry within coastal water columns.

Similarly, nitrate dual isotopic signatures (especially for δ^18^O_NO3_, *p* < 0.05) showed seasonally significant differences between pre- and post-precipitation. Based on indigenous isotopic signatures (Supplementary information Table S4) within traditional end-member ranges^67^, dual isotopic patterns before precipitation within both sections can mainly reflect predominant contributions of natural sources such as soil nitrogen with minor contribution from inorganic fertilizer. Corresponding to POM sources (e.g., soil OM) infiltrating fish farm influents, nitrate sources before precipitation may mostly contain the predominant soil organic N preserved in the aquifer system. In contrast to the isotopic signatures before precipitation, NO_3_ concentrations continuously increased during accumulated precipitation (> 350 mm; NO_3_: >0.4 mg/L). Under these conditions, both the nitrate isotopic compositions exhibited significant isotopic shifts (Fig. 2b). The relative ^15^N-enrichment of NO_3_ in fish farm influents likely indicates a predominant contribution of anthropogenic N sources (mainly sewage and fertilizer) intruding into aquifer systems. In addition, the δ^18^O_NO3_ values were more enriched in fish farm influents as rainfall accumulated; especially for northern sections, reflecting the partial inflow of atmospheric rainfall originated from high latitudes^68,69^. The DIN concentrations dramatically increased under relatively low salinity conditions (approximately 31 PSU; Supplementary Fig. S4). The increased infiltration of terrestrial-derived N sources during accumulated precipitation may have resulted from the faster recharge of the sub-groundwater. Under such conditions, ^18^O-enriched NO_3_ patterns can indicate an increased infiltration of NH_4_ fertilizer derived from agricultural activities^60^. These properties are consistent with those reported in previous studies on the discharge of sub-groundwater from Jeju Island ^[35, 53; 70]^. Thus, the nutrient conditions in fish farm influents may be closely related to the fluctuation of terrestrial-derived groundwater, resulting in the acceleration of anthropogenic NO_3_ contamination during accumulated precipitation^9,58,71^. Given the limited surface discharge on Jeju Island, due to the absence of large streams, fluctuations in sub-groundwater and fish farm influent quality are likely to be directly linked to coastal water quality. In this context, anthropogenic N sources (including NH_4_) released during fish farm operations may significantly contribute to the frequent eutrophication observed in the Jeju coastal environments, where tidal currents are internally circulated along coastal lines within 3 km^72^. The isotopic compositions of POM and nitrate in the effluent were comparable to those in the influent, indicating mixed anthropogenic sources. However, they may also include signals from in-pond biogeochemical processes or seawater dilution (Fig. 2). In this regard, further studies are necessary to monitor the spatiotemporal variations in anthropogenic N release into the coastal regions of Jeju Island.

### Source contribution of particulate and dissolved constituents within fish farm influents

The Bayesian model approach (i.e., MixSIAR) based on the isotopic compositions of POM and NO_3_ was applied to estimate the source contribution of anthropogenic origins intruding into fish farm influents (Fig. 3 and Supplementary Fig. S5). Generally, the uncertainty in the estimates of source contributions can be attributed to the degree of dissimilarity in isotope ratios (i.e., end-member values) between different sources^73,74^. Therefore, we used indigenously isotopic end-members (i.e., inorganic fertilizer, livestock, soil, and septic waste) of POM and NO_3_ collected from Jeju Island. Based on isotopic derived-contribution results, we estimated the quantitative contributions of anthropogenic POM by applying POM-integrated source apportionment to POM that infiltrated into fish-farming influents (Fig. 3). Subsequently, the contribution of anthropogenic POM sources calculated through the mixing model was discriminatively allocated into POM contents, indicating the highest proportions of livestock waste within POM discharge in both sections (northern: 0.1–0.6 mg/L, southern: 0.1–0.5 mg/L). Overall, quantitative variations of inorganic fertilizer (0.1–0.2 mg/L in both sections) intruding into fish farm influents may be heterogeneously influenced by agricultural activities; whereas the distinct intrusion derived from livestock waste may be related to continuous infiltration of anthropogenic OM sources preserved over time^4,8^. With respect to the exponential increase in N production derived from livestock farming and agriculture on Jeju Island (around 1990s), these sources preserved within aquifer systems may be steadily intruding into fish farm influents^4,8,57,70^. Considering that historical records from 1980s to recent years have reported illegal disposal of livestock waste and agricultural runoff from crop production through the highly permeable volcanic structures within Jeju Island^4,8^, these POM sources infiltrating into fish farm influents may potentially contribute to the variations in water quality due to legacy effect. In the near future, further studies should incorporate age dating which will facilitate the precise determination of the anthropogenic POM loading during periodic changes in land-use types.

Overall, the patterns calculated for the NO_3_ fractions were similar to those of the POM fractions (Supplementary Fig. S5). However, the quantitative variations in anthropogenic NO_3_ sources within the fish farm influents were completely different from those in the POM sources. With respect to the time lag in the inflow of anthropogenic sources into particulate and dissolved constituents, substantial variations in anthropogenic NO_3_ sources may be directly influenced by active water-soil interactions, surrounded by different land-use patterns^54,56,75^. These properties suggest that the discriminative land-use types on Jeju Island may alter the substantial intrusion of anthropogenic NO_3_ sources into fish farm influents. Various anthropogenic N sources (e.g., manure, chemical fertilizer, and septic waste) may discriminatively infiltrate the complex aquifer system, including historical NO_3_^−^ contamination within the unsaturated and saturated zones of Jeju Island^4,8,68^. Notably, distinct apportionments of anthropogenic N sources may interact closely with the degree of permeability within bedrock formations. As legacy effects, anthropogenic NO_3_ sources may respond seasonally to an increase in N loading in fish farm influents^8,9,74^. In contract to the southern sections, substantial variations of the anthropogenic NO_3_ sources infiltrated within the northern sections appears to be more directly influenced by permeable volcanic structures, which facilitate a faster and more direct transport of anthropogenic sources into the subsurface aquifer systems compared to conventional recharge processes^8,9^. These processes suggest that continuous heavy precipitation may accelerate the leaching of N (soil and septic waste) from deep soil layers to groundwater and has a larger influence on the increasing NO_3_ concentrations in fish farm influents. In contrast, intermittent heavy precipitation carries nitrogen (livestock waste and chemical fertilizer) from shallow soil through fast flow. In this respect, we propose that a systematic assessment of groundwater fluctuations is important to effectively managing the water quality of fish farm influents. With respect to the quantitative assessment of anthropogenic NO_3_ sources through isotopic approaches, we excluded biogeochemical N processes (e.g., ammonification, nitrification, and denitrification) in this study because of the negligible correlations (R^2^ = 0.1 and *p* > 0.05 for both sections) between NO_3_^−^ concentrations and their isotopic compositions. Generally, if the denitrification process is actively occurring, both parameters (NO_3_^−^ concentrations and δ^15^N_NO3_) indicate a negative correlation as a result of the preferential consumption of ^14^N in the nitrate. In such conditions, δ^15^N_NO3_ and δ^18^O_NO3_ show a relatively positive linear correlation (δ^15^N_NO3_ and δ^18^O_NO3_ ratio ranged 1:1 ~ 1:2) within dual isotopic plots. Although microbial processes (e.g., denitrification) were excluded due to negligible correlation between NO₃⁻ and isotope values, potential fractionation effects in longer residence zones should be considered in future studies, particularly, in saturated and low DO environments. Considering that excess NO_3_^−^ contamination on Jeju Island may intensify due to the infiltration of anthropogenic NO_3_^−^ sources into aquifer systems^4,8,68,70^, further studies on the complex interaction within the NO_3_^−^ cycles under excess N conditions are necessary to manage the water quality of rearing water within fish farm systems.

Based on the quantitative contributions of particulate and dissolved constituents within fish farm influents, we confirmed the spatiotemporally heterogeneous intrusion of anthropogenic sources during fish farm operations. In particular, according to accumulated precipitation, the shift in POM and NO_3_^−^ isotopic compositions indicated spatiotemporal variations in legacy pollutant source inflow, which may be interconnected to land-use patterns. In this study, we focused on the deterioration of influent water quality, which may significantly impact fish culturing production and coastal seawater quality, with respect to the anthropogenic POM and nitrate sources discharged from Jeju groundwater aquifers. Actually, the effluent water in this study represented relatively N- and P-rich conditions and showed a similar POM and nitrate isotopic composition to the influent water. These findings suggest that deterioration of groundwater and influent water could simultaneously impact the coastal region. To mitigate the deterioration of water quality in the fish farm systems and future coastal regions, particularly with increase in precipitation and anthropogenic OM and nutrient influx, integrated management through active and direct remediation should be adopted to eliminate these contaminants. First, for effective source monitoring and regulation, precise source tracking using isotopically distinct end-members (e.g., livestock, septic waste, and inorganic fertilizer) needs to be strengthened to mitigate the unpermitted discharge of livestock waste, untreated effluents, and the indiscriminate use of fertilizers into permeable volcanic aquifers. Previous N contamination cases on Jeju Island (2015^_^2018) have been partly attributed to illegal manure discharge into lava tubes^4,8^. Because infiltration vulnerability varies with the heterogeneous geological formations on Jeju Island, the optimal selection of fish farm locations should be guided by hydrogeological zoning to minimize subsurface contamination risks. Second, as aspects of groundwater quality thresholds (e.g., predefined quality standards: <5.5 mg/L for NO_3_^−^)^4,54^ of Jeju volcanic aquifers, the quality of saline groundwater sources should be evaluated for elevated levels of OM and nutrients. Subsequently, fish farm influents at increased levels can be pretreated or excluded prior to fish farm operations. Finally, considering that the release of mixed anthropogenic contaminants could be continuously recirculated by internal current retention near the Jeju coastal regions, applying wastewater treatment processes (e.g., denitrifying biofilter) and/or extending the drainage outlets of fish farms further offshore is recommended to prevent self-contamination of fish farm influents. Ultimately, when combined with continuous monitoring and strict regulatory enforcement targeting anthropogenic OM/nutrient-driven water quality deterioration, the resilience of aquaculture operations can be significantly enhanced, supporting the sustainable development of fish farming industries on Jeju Volcanic Island. However, these kinds of strategies rely on environmental perspectives related to water quality in the aquaculture system and coastal regions, which may require a significant financial investment. For realistic management recommendations, effective water quality management strategies must rely on both scientific and economic benefits. In this regard, considering both perspectives, further research on management techniques and financial cost is needed.

## Conclusions

Spatiotemporal variations in hydrochemical properties within fish farm influents indicate that accumulated precipitation may directly alter groundwater-seawater interactions, reflecting the increased infiltration of anthropogenic POM (e.g., livestock and inorganic fertilizer) and NO_3_ sources (e.g., livestock and septic waste). Following this, Bayesian mixing model analysis revealed that POM was predominantly released from livestock (39 ± 17%) and soil OM (31 ± 16%), whereas NO₃⁻ mainly originated from soil N (67 ± 7%), with additional contributions from livestock (18 ± 10%) and septic waste (9 ± 4%). Notably, the temporal variation in isotopic compositions during precipitation events indicated the dynamic mobilization of legacy pollutants stored in soil and aquifer systems. Meanwhile, the opposite trends in the quantitative fluxes of POM and NO₃⁻ where POM inputs were more responsive during early rainfall stages (0.5 ± 0.4 mg/L) and NO₃⁻ concentrations increased (1.3 ± 0.9 mg/L) progressively with cumulative precipitation, highlighting the distinct transport pathways of organic and inorganic contaminants. These findings suggest that land-use patterns, precipitation intensity, and geological heterogeneity jointly regulate the vulnerability of aquaculture systems to anthropogenic pollution. To overcome the issue of water quality deterioration in aquaculture influents, regulatory efforts at the terrestrial boundary (e.g., stricter control of livestock waste disposal and fertilizer use in surrounding catchments) should be considered as a priority. Further, to mitigate the degradation of water quality in land-based fish farms and coastal regions, systematic stepwise strategies are necessary for determining scientific monitoring and techniques. In addition, incorporating critical subsurface geological parameters into the hydrogeological zoning will enhance site-specific vulnerability assessments and inform more effective aquacultural site selection and water quality protection strategies. Consequently, these strategies can support the sustainable operation of aquaculture systems on volcanic islands, thereby ensuring the long-term resilience of freshwater and coastal ecosystems in the face of increasing anthropogenic pressures and climate variability.

## Materials and methods

### Study site

**Fig. 5:**
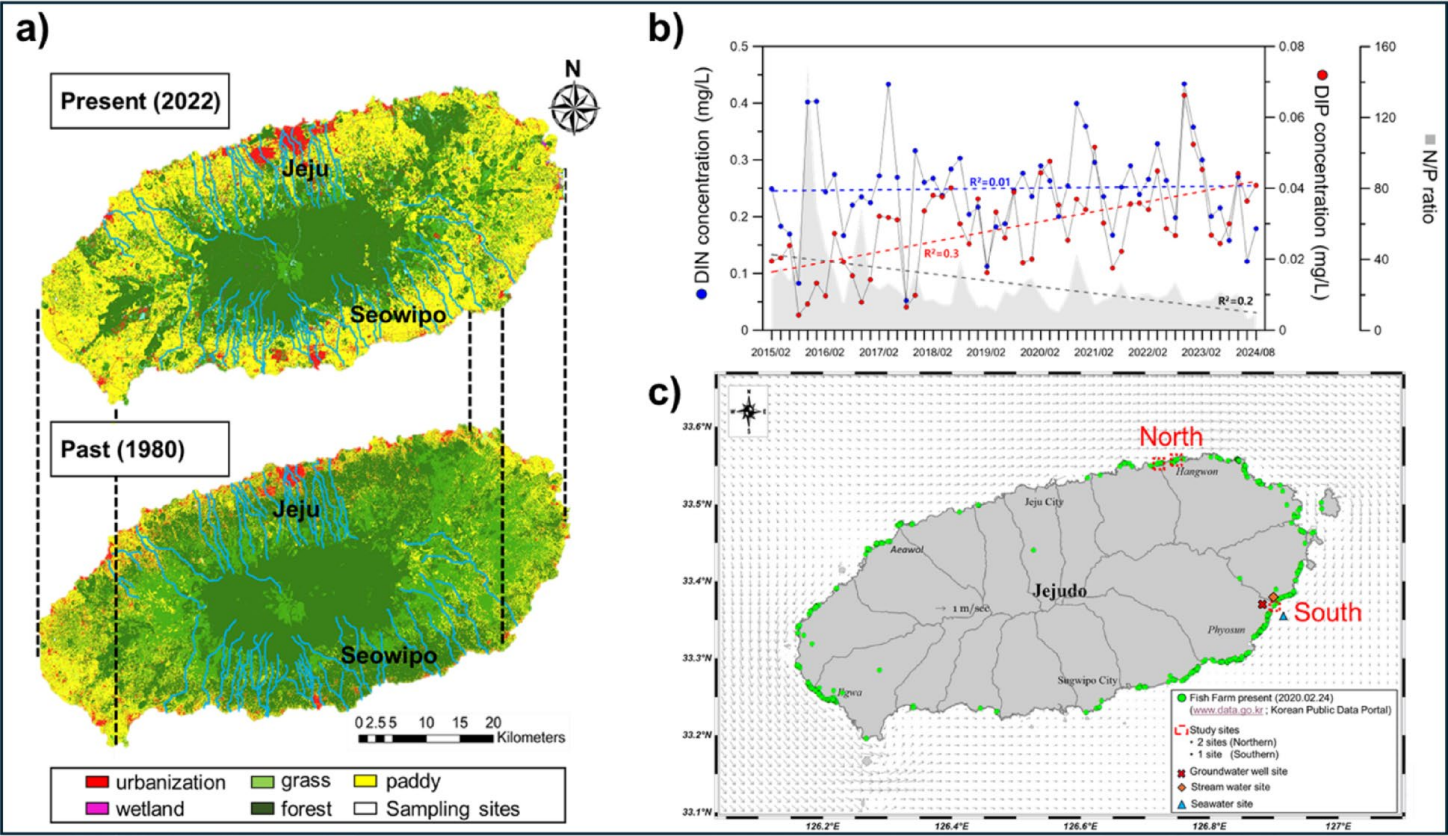
Location map of Jeju Island. Periodical changes of land-use types from 1980 to 2022 (**a**) showing different color categories (red: urbanization, green: grass, yellow: paddy, pink: wetland, olive: forest, “Environmental Geographic Information Service (https://egis.me.go.kr/)” of “Korean Ministry of Environment”). Temporal variations in inorganic nutrients (N and P) and their elemental ratios (2015 to 2024, “Marine Environmental Information Portal; https://www.meis.go.kr/” of “Korean Ministry of Oceans and Fisheries”) (**b**). Study area (**c**) with locations (red dash boxes) of fish farm aquaculture (green circles). The black arrows indicate the direction of coastal currents near Jeju Island. All map figures were treated and represented by ArcGIS (Version 10.2) software and Delft 3D program (Version. 4.06.01).

Jeju Island, a volcanic island located off the southern coast of the Korean Peninsula (latitude; 33.19–33.56 N, longitude; 126.15E-126.97E), has an area of 1,847 km^2^ and over 670,000 a population. The distribution of land-use types within Jeju Island has changed significantly over the past decades (1980–2022) owing to anthropogenic activities (e.g., urbanization and agricultural expansion) (Fig. 5a, Environmental Geographic Information Service; https://egis.me.go.kr/). These anthropogenic developments are primarily concentrated along coastal regions, leading to a substantial discharge of pollutants (e.g., livestock waste, manure, and synthetic fertilizers) into coastal regions^4,8,15–17^. Jeju Island is characterized by high precipitation and permeable volcanic rock formations (e.g., clinker, lava tubes, and tunnels; Supplementary Fig. S1), which facilitate the infiltration of rainfall into subsurface layers of the island^4^. Owing to these hydrogeological characteristics, nearly 50% of the precipitation reaches the aquifer system, with a recharge rate of approximately 46%^8^. Under these hydrogeological conditions, the difference in geographical features, based on Mt. Halla (73 km east to west and 41 km north to south), and climatology (seasonal wind direction and atmospheric pressure) can cause a relatively higher precipitation rate in the southeastern Sect^76^. Furthermore, this discriminative precipitation pattern between geographical sections may induce distinct hydrological responses in the groundwater systems within the northern and southern Sect^77^.

Inland aquaculture is a central business of Jeju’s aquaculture sector, contributing over 90% of the food production. The complete marine enclosure and internal circulation of tidal current conditions in Jeju supported the growth of numerous land-based fish farms^64,78^. Under these conditions, the relatively stable temperature of saline groundwater throughout the year (14–17 ℃, pH 7.2) makes it a suitable rearing water for a flow-through operating system, promoting the development of inland aquaculture systems. Among these fish farms, over 90% of systems are culturing Bastard halibut (Paralichthys olivaceus, flatfish), which represent typical production scales (around 120 tons per year) and geographic distribution across the island. In Jeju Island, around 360 aquaculture farms are fully operated in most coastal areas (east; 44%, south; 30%, north; 19%, west; 7%), which are most concentrated in north eastern (around 44%) part (www.data.go.kr, “Korean Public Data Portal”). These fish farms operate based on saline groundwater, and generally administer feed supply one to three times daily, which could make OM and nutrient concentration of effluent water fluctuate sharply on short time scales^79^.

Under such conditions, long-term variations in nutrient concentrations (e.g., dissolved inorganic nitrogen [DIN] and dissolved inorganic phosphate [DIP]) have shown seasonally distinct fluctuations in coastal seawater, which are closely linked to submarine groundwater and fish farm effluent discharge (Fig. 5b, “Marine Environmental Information Portal; https://www.meis.go.kr/” of “Korean Ministry of Oceans and Fisheries”)^4,17^. Especially, DIN concentrations in seawater remained relatively stable (0.1–0.4 mg/L), while DIP concentrations exhibited a significant increasing trend (R² = 0.3, *p* < 0.01). Based on these trends, the DIN: DIP elemental ratio showed a decreasing trend over the past decade (R^2^ = 0.2, *p* < 0.01). Furthermore, the internal circulation of tidal currents, calculated to range between 0.1 and 0.9 m/s through Delft 3D program (Ver. 4.06.01, Deltarres, Netherlands) showed the restricted discharge of surface and groundwater into coastal area of Jeju Island (Fig. 5c), leading to the negative feedback mechanism associated with the self-contamination of anthropogenic sources occurring at the terrestrial-marine interface^13,72,78^.

### Environmental sample collecting

To compare the effect of heavy precipitation on nitrate release through sub-groundwater discharge in influent and effluent water within fish farms, water samples were separated into pre- (May 2022) and post-precipitation periods (June to September 2022). Daily and accumulated precipitation data for east northern and southern regions of Jeju Island were collected from the Korean Meteorological Administration Weather Data Service (open MET Data portal, https://data.kma.go.kr). Water samples (i.e., influents and effluents) were spatiotemporally collected from three land-based aquaculture system sites in Jeju Island (northern section: two sites; southern section: one site; Fig. 5c). Among regional sections, we selected eastern section of Jeju Island, where inland aquaculture systems are concentrated. Also, to compare the precipitation effect on influent water, we divided study section into northern and southern regions. To identify the specific POM and nitrate sources in the study area, local end-member samples (i.e., terrestrial soil, inorganic fertilizer, septic waste, and livestock waste) were simultaneously collected from around inland fish farms in both the northern and southern sections. For the nitrate isotope end-member, the collected source samples were soaked and shaken in deionized water for 24 h using a vertical shaker to extract nitrate.

To collect particulate OM and dissolved constituents, the influent water samples were filtered through a 0.45 μm GF-5 filter (diameter: 125 mm, MACHEREY-NAGEL, Germany) in the laboratory. Together with in situ hydrophysical properties (i.e., water temperature, salinity, pH, and dissolved oxygen [DO]) of influents within land-based aquaculture systems, one of the filtered samples was freeze-dried and weighed to calculate the concentration of total suspended matter. Other filtered samples were stored at -20 °C prior to geochemical analysis. Finally, the filtrates were treated with HgCl_2_ in 40 mL amber vials to obtain dissolved organic carbon (DOC) concentrations. Samples were stored at 4 °C prior to analysis. For inorganic nutrient (DIN: sum of NO_2_^−^, NO_3_^−^, and NH_4_^+^; DIP: PO_4_^3−^) concentration and nitrate dual isotopic compositions (δ^15^N_NO3_, δ^15^N_NO3_), fraction of filtrate samples were stored at -40 °C. For major elements (Na^+^, K^+^, Ca^2+^, and Mg^2+^), fraction of filtrate sample were acidified to decrease pH < 2 by adding nitric acid (HNO_3_) and stored at 4 °C before analysis, while fraction for anion (Cl^−^, SO_4_^2−^, and HCO_3_^−^) concentration sample were stored at -40 °C with no acidification. All samples were collected in duplicate or triplicate to ensure analytical reliability. Further, all data reported in this study are presented as averages ± 1 standard deviation.

### Hydrochemical analysis

The concentrations of particulate organic carbon (POC) and particulate nitrogen (PN), and freeze-dried filtered sample were measured using an elemental analyzer (ElementarVario MICRO CUBE, Elementar, Langenlbold, Germany). For POC concentration analysis, inorganic carbon was removed from the filtered sample using 12 M HCl under a fume hood. After the fume treatment, the filtered sample was neutralized using NaOH pellets. For the PN concentration, the samples were analyzed without any prior treatment. The analytical precisions (± 1σ standard deviation) estimated using IAEA-CH-3 and IAEA-N-1 (International Atomic Energy Agency, Vienna, Austria) were ± 0.2% and ± 0.4% for POC and PN, respectively.

DOC and DTN concentrations were measured by oxidative combustion-chemiluminescence using a total organic carbon analyzer (TOC-L CPH/CPN, Shimazu, Kyoto, Japan). The analytical precision (± 1σ standard deviation) determined by measuring reference materials (surface seawater reference, Dennis Hansell of Marine and Atmospheric Sciences, University of Miami, USA) was less than ± 0.2% and ± 0.3%. Nutrient concentrations (NO_3_^−_^N, NH_4_^+^-N, PO_4_^3−^P, and SiO_4_^2−^_Si) were analyzed using a QuAAtro Auto-Analyzer (Seal Analytical Ltd., Southampton, UK). The limits of quantification were 0.1, 0.2, and 0.02 µmol L^− 1^ for NO_3_-N, NH_4_-N, and PO_4_-P, respectively. The analytical precision was less than ± 10% for nutrients. The bicarbonate (HCO_3_^−^) concentration was determined by potentiometric acid titration to pH 4.5 using 0.01 N HCl with an automatic titrator (T50A, Mettler–Toledo, Columbus, OH, USA). Concentrations of anions (Cl^−^ and SO_4_^2−^) were measured using Ion Chromatography (IC, HIC-ESP, Shimadzu) and major elements (Na^+^, K^+^, Ca^2+^, and Mg^2+^) for an Inductively Coupled Plasma Optical Emission Spectroscopy (ICP-OES, Optima 7000DV, PerkinElmer) installed at the Integrated Analytical Center for Earth and Environmental Sciences of Pukyong National University. The analytical precision for ICP-OES was tested using certified reference material (International Association for Physical Sciences of the Ocean, IAPSO standard seawater) and less than ± 4% for standard.

### Stable isotopic composition analysis

The isotopic compositions of POC and PN (δ^13^C_POC_ and δ^15^N_PN_) were analyzed simultaneously with POC and PN concentrations using an elemental analyzer connected with isotope ratio mass spectrometry (VisIONElementar, Hesse, Germany). The isotope ratio was expressed in delta (δ) notation relative to Vienna Pee-Dee Belemnite (VPDB) and atmospheric N_2_ for carbon and nitrogen, respectively. Instrumental condition and analytical precision were monitored, using certified standard material IAEA-CH-3 (δ^13^C = -24.7‰) and IAEA-N-1 (δ^15^*N* = 0.4‰). Analytical precisions (± 1σ standard deviation) were less than ± 0.1‰ and ± 0.2‰ for δ^13^C_POC_ and δ^15^N_PN_ respectively.

The isotopic compositions of nitrate were analyzed using a bacterial denitrification method^80^ with trace gas analyzer-isotope ratio mass spectrometry (TG-IRMS; Trace gas-Isoprime 100, Langenlbold, Germany) at the National Institute of Fisheries Science. All stable isotope values were expressed by δ notation relative to the Air and Vienna Standard Mean Ocean Water (VSMOW) standard for nitrogen and oxygen, respectively. International reference materials (IAEA-NO-3, USGS34, and USGS35) were used for data calibration and instrumental monitoring. All water samples were measured in triplicate. Analytical precision (± 1σ standard deviation) was less than ± 0.2‰ and ± 0.5‰ for δ^15^N_NO3_ and δ^18^O_NO3_, respectively.

### Statistical analysis

Pearson correlation analysis was conducted using SPSS software (IBM Analytics, Chicago, USA, Version 29) to identify the correlations among the hydrochemical parameters such as temperature, salinity, DO, pH, POC, PN, DOC, DIN, DIP, and dissolved silicate (DSi, SiO_4_^2−^). Significant correlations between these parameters were indicated by the Pearson correlation coefficient (*r*) along with the corresponding significance level (*p*). In addition, linear correlations between Cl^−^ and major ions (SO_4_^2−^, HCO_3_^−^, Na^+^, K^+^, Ca^2+^, and Mg^2+^) were analyzed using Grapher software (Golden Software, LLC, Colorado, USA, Version 24). Furthermore, 95% confidence intervals for the linear regression lines were calculated and visualized using Grapher software.

### Bayesian mixing model

Based on the variations of δ^13^C_POC_, δ^15^N_PN_, δ^15^N_NO3_, and δ^15^N_NO3_ in influent and effluent water samples, the proportional contribution of POM and nitrate sources was estimated using Bayesian stable isotope mixing model (Bayesian Mixing Model in R; MixSIAR, version 3.1.10)^81^. In this study, local end-member sources (soil, inorganic fertilizer, livestock, and septic waste) were collected to determine the relative contribution of each source transported in the fish farm systems. In the MixSIAR model, these local end-members and fish farm water were assigned as “Source” and “Mixture,” respectively. With respect to geochemical reactions (i.e., source input and consumption) within the sub-groundwater, the isotopic fractionation derived from nitrification and denitrification was excluded from this study (Supplementary Fig. S4).

## Supplementary Information

Below is the link to the electronic supplementary material.

Supplementary Material 1

Supplementary Material 2

## Data Availability

All data generated or analysed during this study are included in this published article and its supplementary information files (Figures and Tables). Also, additional information (i.e. extra dataset) in this study used during the current study available from the corresponding author on reasonable request.
