# Supplementary material for: Hydrochemical and isotopic signatures of groundwater infiltration and legacy nitrogen discharge within Jeju Island aquaculture systems
Source: Sci Rep. 2026 Jan 6;16:703. doi: 10.1038/s41598-025-26436-9 (PMC12780187; doi:10.1038/s41598-025-26436-9)
Supplement: Supplementary file 1 — Supplementary Material 1 [file 41598_2025_26436_MOESM1_ESM.docx]

**Table S1**. Physicochemical properties of fish farm influent and effluent water samples from the east northern and southern regions of Jeju Island.

| **Date** | | **Region** | **Type** | **Temperature** | **Salinity** | **DO** | **pH** | **POC** | **PN** | **DOC** | **DIN** | **NO_3_^-^** | **NO_2_^-^** | **NH_4_^+^** | **PO_4_^3-^** | **SiO_4_^2-^** | **δ^13^C_POC_** | **δ^15^N_PN_** | **δ^15^N_NO3_** | **δ^18^O_NO3_** |
| --- | --- | --- | --- | --- | --- | --- | --- | --- | --- | --- | --- | --- | --- | --- | --- | --- | --- | --- | --- | --- |
| Year | Month. Day |  |  | ^o^C | psi | mg/L |  | mg/L | mg/L | mg/L | mg/L | mg/L | mg/L | mg/L | mg/L | mg/L | ‰ | ‰ | ‰ | ‰ |
| 2022 | 05. 11 | Northern | *IF | 17.60 | 33.10 | 7.49 | 7.89 | 0.20 | 0.01 | 0.53 | 0.24 | 0.24 | 0.00 | 0.00 | 0.07 | 5.14 | -30.14 | 2.77 | 6.78 | -3.90 |
|  | 05. 11 |  | IF | 17.98 | 32.63 | 5.33 | 7.96 | 0.16 | 0.02 | 0.54 | 0.16 | 0.15 | 0.00 | 0.00 | 0.02 | 2.11 | -24.83 | 2.87 | 6.78 | -2.83 |
|  | 06. 24 |  | IF | 18.87 | 33.83 | 8.42 | 7.93 | 1.09 | 0.13 | 0.87 | 0.36 | 0.31 | 0.00 | 0.04 | 0.10 | 5.90 | -22.83 | 4.52 | - | - |
|  | 08. 01 |  | IF | 20.30 | 32.30 | 8.04 | 8.01 | 0.39 | 0.36 | 0.62 | 0.26 | 0.03 | 0.00 | 0.23 | 0.05 | 4.78 | -21.70 | 6.33 | 17.96 | -1.39 |
|  | 08. 01 |  | IF | 26.10 | 29.70 | 6.41 | 8.19 | 0.28 | 0.01 | 1.19 | 0.54 | 0.01 | 0.00 | 0.52 | 0.01 | 0.94 | -21.92 | 4.00 | 7.89 | 1.71 |
|  | 08. 18 |  | IF | 20.75 | 32.60 | 8.27 | 8.02 | 0.42 | 0.22 | 0.84 | 0.30 | 0.13 | 0.01 | 0.16 | 0.03 | 1.57 | -24.01 | 2.45 | 6.45 | 6.10 |
|  | 08. 18 |  | IF | 26.30 | 29.80 | 6.14 | 7.92 | 0.24 | 0.14 | 1.51 | 0.10 | 0.09 | 0.00 | 0.01 | 0.01 | 0.27 | -22.42 | 5.55 | 6.10 | 2.00 |
|  | 09. 06 |  | IF | 18.20 | 34.10 | 10.02 | 7.74 | 0.14 | 0.26 | 0.56 | 0.18 | 0.11 | 0.00 | 0.06 | 0.02 | 0.78 | -21.98 | 1.42 | 6.70 | 7.10 |
|  | 09. 06 |  | IF | 23.70 | 32.90 | 6.75 | 7.95 | 0.47 | 0.31 | 2.40 | 1.11 | 0.60 | 0.02 | 0.49 | 0.11 | 4.87 | -22.37 | 7.66 | 7.00 | 9.80 |
|  | 09. 21 |  | IF | 19.45 | 33.70 | 8.22 | 8.10 | 0.15 | 0.06 | 0.67 | 0.87 | 0.77 | 0.01 | 0.09 | 0.25 | 21.24 | -26.48 | 1.76 | 5.94 | -0.58 |
|  | 09. 21 |  | IF | 22.20 | 32.70 | 7.28 | 8.26 | 0.27 | 0.20 | 1.18 | 2.05 | 0.65 | 0.03 | 1.37 | 0.15 | 5.46 | -20.57 | 4.73 | 6.10 | 0.30 |
|  | 05. 11 |  | **EF | 17.20 | 33.20 | 5.94 | 7.64 | 0.39 | 0.04 | 0.68 | 0.40 | 0.20 | 0.01 | 0.20 | 0.08 | 4.61 | -23.32 | 2.54 | 6.72 | -4.20 |
|  | 05. 11 |  | EF | 17.55 | 33.45 | 7.14 | 7.86 | 0.33 | 0.02 | 0.74 | 0.32 | 0.13 | 0.00 | 0.18 | 0.05 | 1.51 | -23.20 | 5.57 | 6.87 | -2.25 |
|  | 05. 10 | Southern | IF | 17.42 | 32.83 | 6.58 | 7.99 | 0.19 | 0.01 | 0.70 | 0.26 | 0.25 | 0.00 | 0.01 | 0.03 | 1.92 | -23.92 | 0.14 | 5.56 | -4.41 |
|  | 05. 10 |  | IF | 19.15 | 33.59 | 6.44 | 7.73 | 1.30 | 0.20 | 1.67 | 0.56 | 0.13 | 0.00 | 0.43 | 0.07 | 1.09 | -19.60 | 5.41 | 2.65 | 6.14 |
|  | 06. 24 |  | IF | 18.03 | 32.68 | 5.85 | 7.81 | 0.19 | 0.01 | 0.70 | 0.60 | 0.59 | 0.00 | 0.01 | 0.08 | 4.17 | -27.03 | - | 2.95 | 0.68 |
|  | 08. 01 |  | IF | 19.33 | 31.65 | 4.49 | 7.94 | 0.10 | 0.31 | 0.43 | 0.22 | 0.05 | 0.00 | 0.16 | 0.05 | 3.08 | -24.45 | 1.32 | 6.60 | 1.10 |
|  | 08. 18 |  | IF | 19.70 | 30.60 | 4.75 | 7.84 | 0.11 | 0.25 | 0.73 | 0.38 | 0.33 | 0.00 | 0.05 | 0.04 | 2.07 | -25.78 | 0.99 | 6.80 | 0.48 |
|  | 09. 06 |  | IF | 20.33 | 29.60 | 4.62 | 7.77 | 0.13 | 0.13 | 0.59 | 0.45 | 0.42 | 0.00 | 0.02 | 0.04 | 2.19 | -24.55 | 2.65 | 5.49 | -3.64 |
|  | 09. 21 |  | IF | 20.55 | 31.58 | 5.01 | 8.13 | 0.11 | 0.23 | 0.57 | 2.06 | 1.97 | 0.00 | 0.09 | 0.23 | 15.26 | -28.57 | 2.51 | 6.18 | -1.45 |
|  | 05. 10 |  | EF | 21.35 | 33.61 | 6.83 | 7.80 | 0.60 | 0.08 | 1.88 | 0.56 | 0.14 | 0.00 | 0.42 | 0.11 | 1.24 | -20.44 | 4.90 | 4.05 | 7.02 |
|  |  |  |  |  |  |  |  |  |  |  |  |  |  |  |  |  |  |  |  |  |
| 2024 | 06. 26 | Northern | IF | 19.73 | 25.74 | 7.62 | 8.01 | 0.19 | 0.04 | 0.70 | 1.18 | 1.17 | 0.00 | 0.01 | 0.04 | 3.48 | -22.11 | 3.21 | 4.49 | 3.11 |
|  | 07. 02 |  | IF | 19.77 | 26.20 | 8.11 | 7.97 | 0.18 | 0.02 | 0.67 | 1.27 | 1.26 | 0.00 | 0.01 | 0.05 | 3.47 | -22.19 | 1.66 | 3.83 | 0.90 |
|  | 07. 02 |  | IF | 19.57 | 30.73 | 7.73 | 8.04 | 0.11 | 0.02 | 0.77 | 0.48 | 0.47 | 0.00 | 0.00 | 0.04 | 2.84 | -20.85 | 2.39 | - | - |
|  | 07. 02 |  | IF | 19.30 | 25.67 | 8.55 | 8.09 | 0.07 | 0.02 | 0.75 | 1.54 | 1.54 | 0.00 | 0.01 | 0.04 | 3.30 | -22.19 | 3.49 | - | - |
|  | 07. 03 |  | IF | 22.40 | 30.70 | 7.77 | 8.08 | 0.10 | 0.02 | 1.08 | 0.13 | 0.10 | 0.00 | 0.03 | 0.02 | 1.19 | -19.91 | 2.18 | - | - |
|  | 07. 03 |  | IF | 17.40 | 33.80 | 8.51 | 7.80 | 0.26 | 0.01 | 0.41 | 0.18 | 0.18 | 0.00 | 0.00 | 0.06 | 5.01 | -24.67 | -0.89 | - | - |
|  | 07. 09 |  | IF | 20.70 | 24.07 | 8.37 | 8.16 | 0.14 | 0.04 | 0.75 | 1.32 | 1.29 | 0.00 | 0.03 | 0.05 | 3.99 | -19.21 | 3.10 | 4.32 | 0.72 |
|  | 07. 09 |  | IF | 22.50 | 30.10 | 7.65 | 8.10 | 0.10 | 0.02 | 1.06 | 0.15 | 0.13 | 0.00 | 0.02 | 0.02 | 1.62 | -21.24 | 5.71 | - | - |
|  | 07. 09 |  | IF | 17.60 | 33.90 | 8.33 | 7.80 | 0.02 | 0.01 | 0.57 | 0.19 | 0.18 | 0.00 | 0.00 | 0.06 | 5.25 | -23.16 | 6.23 | - | - |
|  | 07. 16 |  | IF | 23.00 | 30.00 | 7.56 | 8.08 | 0.07 | 0.02 | 0.91 | 0.16 | 0.14 | 0.00 | 0.02 | 0.02 | 1.59 | -21.65 | 0.49 | - | - |
|  | 07. 17 |  | IF | 20.70 | 24.90 | 8.08 | 8.06 | 0.37 | 0.03 | 0.79 | 1.30 | 1.26 | 0.00 | 0.04 | 0.04 | 3.75 | -22.62 | 2.47 | - | - |
|  | 07. 17 |  | IF | 17.30 | 33.90 | 7.81 | 5.72 | 0.04 | 0.01 | 0.30 | 0.17 | 0.17 | 0.00 | 0.00 | 0.06 | 5.07 | -25.67 | - | - | - |
|  | 06. 27 | Southern | IF | 18.93 | 29.77 | 8.18 | 8.04 | 0.19 | 0.04 | 0.79 | 0.45 | 0.45 | 0.00 | 0.00 | 0.03 | 1.96 | -22.36 | 3.12 | 4.72 | 4.16 |
|  | 07. 02 |  | IF | 19.40 | 29.67 | 8.43 | 8.03 | 0.06 | 0.01 | 0.69 | 0.41 | 0.41 | 0.00 | 0.00 | 0.02 | 1.55 | -22.29 | 1.49 | 5.14 | 3.32 |
|  | 07. 09 |  | IF | 20.13 | 29.67 | 8.52 | 8.03 | 0.08 | 0.02 | 0.65 | 0.47 | 0.46 | 0.00 | 0.00 | 0.03 | 2.01 | -22.09 | 1.02 | 6.75 | 5.00 |
|  | 07. 16 |  | IF | 19.67 | 29.97 | 8.16 | 8.01 | 0.06 | 0.01 | 0.63 | 0.43 | 0.42 | 0.00 | 0.01 | 0.03 | 1.96 | -23.69 | 1.08 | - | - |

^*^IF: fish farm influent water

^**^EF: fish farm effluent water

**Table S2**. Major cation and anion concentrations in fish farm influent and effluent, groundwater well, stream, and seawater samples in the east northern, and southern regions of Jeju Island.

| **Date** | | **Region** | **Type** | **Mg^2+^** | **Ca^2+^** | **Na^+^** | **K^+^** | **Cl^-^** | **SO_4_^2-^** | **HCO_3_^-^** |
| --- | --- | --- | --- | --- | --- | --- | --- | --- | --- | --- |
| Year | Mounth. Day |  |  | mg/L | mg/L | mg/L | mg/L | mg/L | mg/L | mg/L |
| 2022 | 05. 11 | Northern | IF | 997.0 | 309.5 | 8295.3 | 321.0 | 12634.6 | 1862.2 | 113.3 |
|  | 05. 11 |  | IF | 946.7 | 278.6 | 7384.0 | 289.6 | 15950.3 | 2248.2 | 103.5 |
|  | 06. 24 |  | IF | 1327.7 | 391.4 | 10245.6 | 409.2 | 19317.4 | 2636.6 | 131.7 |
|  | 08. 01 |  | IF | 1123.3 | 356.2 | 10090.3 | 379.8 | 16322.7 | 2426.6 | 78.7 |
|  | 08. 01 |  | IF | 1195.6 | 380.7 | 10709.2 | 400.4 | 17333.5 | 2589.1 | 81.2 |
|  | 08. 18 |  | IF | 1418.2 | 453.9 | 12604.6 | 471.0 | 20536.8 | 2963.6 | 106.3 |
|  | 08. 18 |  | IF | 1411.9 | 444.7 | 12661.1 | 465.8 | 20332.2 | 2956.9 | 108.6 |
|  | 09. 06 |  | IF | 1532.1 | 490.9 | 14417.3 | 537.6 | 22651.3 | 3220.2 | 108.8 |
|  | 09. 06 |  | IF | 439.6 | 130.1 | 3697.3 | 145.2 | 7473.3 | 750.7 | 18.7 |
|  | 09. 21 |  | IF | 1605.2 | 507.6 | 14550.8 | 545.6 | 22758.7 | 3210.4 | 131.3 |
|  | 09. 21 |  | IF | 1605.8 | 505.3 | 14609.7 | 533.9 | 27541.9 | 3239.4 | 142.4 |
|  | 05. 11 |  | EF | 811.9 | 246.5 | 6614.6 | 259.6 | 15218.4 | 2422.1 | 94.3 |
|  | 05. 11 |  | EF | 1123.8 | 332.0 | 8861.8 | 349.8 | 17582.0 | 2437.7 | 102.5 |
|  | 05. 10 | Southern | IF | 1016.1 | 305.3 | 8063.2 | 312.1 | 16262.4 | 2404.9 | 89.4 |
|  | 05. 10 |  | IF | 948.3 | 302.3 | 7950.1 | 310.3 | 18091.9 | 2483.3 | 143.1 |
|  | 06. 24 |  | IF | 1253.7 | 373.0 | 9787.1 | 389.8 | 17140.2 | 2431.0 | 125.3 |
|  | 08. 01 |  | IF | 1114.6 | 359.6 | 10160.0 | 380.3 | 16460.6 | 2469.5 | 81.0 |
|  | 08. 18 |  | IF | 1376.4 | 438.4 | 12509.0 | 471.1 | 20175.4 | 2920.4 | 99.3 |
|  | 09. 06 |  | IF | 1240.6 | 393.9 | 11317.0 | 425.1 | 17536.8 | 2457.7 | 103.6 |
|  | 09. 21 |  | IF | 1517.9 | 482.9 | 13915.2 | 519.7 | 22601.3 | 3180.8 | 129.5 |
|  | 05. 10 |  | EF | 744.1 | 254.4 | 6882.2 | 261.5 | 16137.0 | 2458.5 | 119.4 |
|  |  |  |  |  |  |  |  |  |  |  |
|  | 05.12 | Southern | ^*^GW | 4.1 | 3.2 | 30.9 | 10.3 | 43.2 | 5.7 | 27.7 |
|  | 05.12 |  | GW | 98.3 | 30.3 | 846.5 | 37.2 | 1420.6 | 450.5 | 39.2 |
|  | 05.12 |  | GW | 6.9 | 5.8 | 36.5 | 8.6 | 56.8 | 18.3 | 46.0 |
|  | 05.12 |  | Stream | 573.1 | 191.8 | 4771.5 | 180.8 | 7384.6 | 1045.5 | 66.4 |
|  | 05.12 |  | Stream | 614.3 | 180.7 | 4712.0 | 186.9 | 10213.4 | 1566.9 | 119.4 |
|  | 05.12 |  | Stream | 870.3 | 256.0 | 6999.6 | 271.2 | 7160.9 | 1674.7 | 97.3 |
|  | 06.24 |  | Stream | 815.0 | 241.3 | 6333.1 | 255.2 | 11649.9 | 1663.2 | 108.6 |
|  | 08.01 |  | Stream | 24.9 | 25.2 | 118.7 | 19.2 | 183.8 | 67.6 | 55.5 |
|  | 08.01 |  | Stream | 291.6 | 95.6 | 2501.6 | 96.7 | 4047.5 | 791.7 | 39.0 |
|  | 08.18 |  | Stream | 31.2 | 37.2 | 61.4 | 16.6 | 247.6 | 63.8 | 1.0 |
|  | 08.18 |  | Stream | 173.2 | 54.0 | 1495.4 | 61.7 | 2569.2 | 595.7 | -2.8 |
|  | 08.18 |  | Seawater | 1137.5 | 358.1 | 10080.1 | 384.8 | 15929.9 | 2453.3 | 92.4 |
|  | 09.06 |  | Stream | 33.5 | 42.8 | 106.7 | 20.6 | 246.1 | 101.7 | 21.9 |
|  | 09.06 |  | Stream | 76.0 | 23.4 | 667.6 | 30.1 | 1515.9 | 165.9 | 19.0 |
|  | 09.06 |  | Seawater | 1391.2 | 435.7 | 12387.0 | 469.7 | 19083.5 | 2656.8 | 75.2 |
|  | 09.21 |  | Stream | 796.2 | 248.6 | 6984.9 | 264.1 | 12365.6 | 1934.0 | 52.0 |
|  | 09.21 |  | Seawater | 1767.0 | 564.6 | 16184.7 | 588.0 | 24866.0 | 3403.2 | 145.4 |

^*^GW: groundwater well

**Table S3**. Pearson correlation analysis between physical and chemical parameters of fish farm influent in the northern and southern section of Jeju Island.

| **Northern** | **Temperature** | **Salinity** | **DO** | **pH** | **POC** | **PN** | **DOC** | **DIN** | **NO_3_^-^** | **NO_2_^-^** | **NH4** | **PO_4_^3^** | **SiO_4_^2-^** |
| --- | --- | --- | --- | --- | --- | --- | --- | --- | --- | --- | --- | --- | --- |
| **Temperature** | 1 | -0.8** | -0.3 | 0.5 | -0.1 | 0.2 | 0.7** | 0.3 | 0.0 | 0.2 | 0.4 | -0.2 | -0.2 |
| **Salinity** |  | 1 | 0.5 | -0.4 | 0.2 | 0.1 | -0.4 | 0.1 | 0.4 | 0.2 | -0.1 | 0.5 | 0.4 |
| **DO** |  |  | 1 | -0.1 | 0.2 | 0.5 | -0.3 | -0.1 | 0.1 | -0.1 | -0.1 | 0.2 | 0.2 |
| **pH** |  |  |  | 1 | -0.1 | 0.1 | 0.2 | 0.6* | 0.4 | 0.4 | 0.6* | 0.3 | 0.3 |
| **POC** |  |  |  |  | 1 | 0.2 | 0.2 | -0.1 | 0.0 | -0.0 | -0.1 | 0.1 | -0.0 |
| **PN** |  |  |  |  |  | 1 | 0.4 | 0.2 | 0.1 | 0.3 | 0.2 | -0.0 | -0.1 |
| **DOC** |  |  |  |  |  |  | 1 | 0.4 | 0.3 | 0.5 | 0.4 | 0.1 | -0.1 |
| **DIN** |  |  |  |  |  |  |  | 1 | 0.8** | 0.9** | 0.9** | 0.6* | 0.3 |
| **NO_3_^-^** |  |  |  |  |  |  |  |  | 1 | 0.7** | 0.4 | 0.9** | 0.8** |
| **NO_2_^-^** |  |  |  |  |  |  |  |  |  | 1 | 0.8** | 0.6* | 0.3 |
| **NH_4_^+^** |  |  |  |  |  |  |  |  |  |  | 1 | 0.3 | -0.0 |
| **PO_4_^3-^** |  |  |  |  |  |  |  |  |  |  |  | 1 | 0.9** |
| **SiO_4_^2-^** |  |  |  |  |  |  |  |  |  |  |  |  | 1 |

| **Southern** | **Temperature** | **Salinity** | **DO** | **pH** | **POC** | **PN** | **DOC** | **DIN** | **NO_3_^-^** | **NO_2_^-^** | **NH4** | **PO_4_^3^** | **SiO_4_^2-^** |
| --- | --- | --- | --- | --- | --- | --- | --- | --- | --- | --- | --- | --- | --- |
| **Temperature** | 1 | -0.1 | -0.1 | 0.4 | 0.1 | 0.7* | 0.7* | 0.6* | 0.3 | 0.6* | 0.6* | 0.4 | 0.1 |
| **Salinity** |  | 1 | 0.8** | 0.1 | 0.5 | -0.4 | 0.4 | 0.1 | -0.2 | 0.2 | 0.3 | 0.3 | 0.1 |
| **DO** |  |  | 1 | 0.3 | 0.3 | -0.4 | 0.4 | 0.2 | -0.1 | 0.5 | 0.4 | 0.4 | 0.3 |
| **pH** |  |  |  | 1 | -0.4 | 0.3 | -0.1 | 0.7** | 0.6* | 0.5 | 0.4 | 0.6* | 0.6* |
| **POC** |  |  |  |  | 1 | 0.1 | 0.7* | -0.1 | -0.3 | -0.0 | 0.3 | -0.1 | -0.3 |
| **PN** |  |  |  |  |  | 1 | 0.3 | 0.4 | 0.2 | 0.2 | 0.3 | 0.1 | -0.0 |
| **DOC** |  |  |  |  |  |  | 1 | 0.2 | -0.1 | 0.4 | 0.5 | 0.0 | -0.2 |
| **DIN** |  |  |  |  |  |  |  | 1 | 0.8** | 0.6* | 0.6* | 0.7** | 0.5 |
| **NO_3_^-^** |  |  |  |  |  |  |  |  | 1 | 0.1 | -0.0 | 0.8** | 0.7* |
| **NO_2_^-^** |  |  |  |  |  |  |  |  |  | 1 | 0.8** | 0.4 | 0.2 |
| **NH_4_^+^** |  |  |  |  |  |  |  |  |  |  | 1 | 0.2 | -0.1 |
| **PO_4_^3-^** |  |  |  |  |  |  |  |  |  |  |  | 1 | 0.9** |
| **SiO_4_^2-^** |  |  |  |  |  |  |  |  |  |  |  |  | 1 |

* *p*<0.05

** *p*<0.01

**Table S4**. Stable isotopic compositions of particulate organic matter and nitrate end-members.

| **Source sample** | **δ^13^C_POC_ (‰)** | | **δ^15^N_PN_ (‰)** | | **δ^15^N_NO3_ (‰)** | | **δ^18^O_NO3_ (‰)** | |
| --- | --- | --- | --- | --- | --- | --- | --- | --- |
|  | Average±SD | n | Average±SD | n | Average±SD | n | Average±SD | n |
| Terrestrial soil | -29.4±0.7 | 5 | 2.4±4.6 | 5 | 1.5±1.7 | 5 | -2.0±1.2 | 5 |
| Inorganic fertilizer | -26.0±1.5 | 5 | -3.2±1.2 | 5 | 3.9±1.2 | 5 | 9.4±0.6 | 5 |
| Septic waste | -24.1±0.6 | 5 | 9.7±0.3 | 5 | 13.3±0.6 | 5 | 0.6±0.6 | 5 |
| Livestock | -19.0±1.2 | 5 | 8.0±0.3 | 5 | 17.6±3.7 | 5 | 6.4±0.6 | 5 |
