# Supplementary material for: Hydrochemical and isotopic signatures of groundwater infiltration and legacy nitrogen discharge within Jeju Island aquaculture systems
Source: Sci Rep. 2026 Jan 6;16:703. doi: 10.1038/s41598-025-26436-9 (PMC12780187; doi:10.1038/s41598-025-26436-9)
Supplement: Supplementary file 2 — Supplementary Material 2 [file 41598_2025_26436_MOESM2_ESM.pptx]

## Slide 1
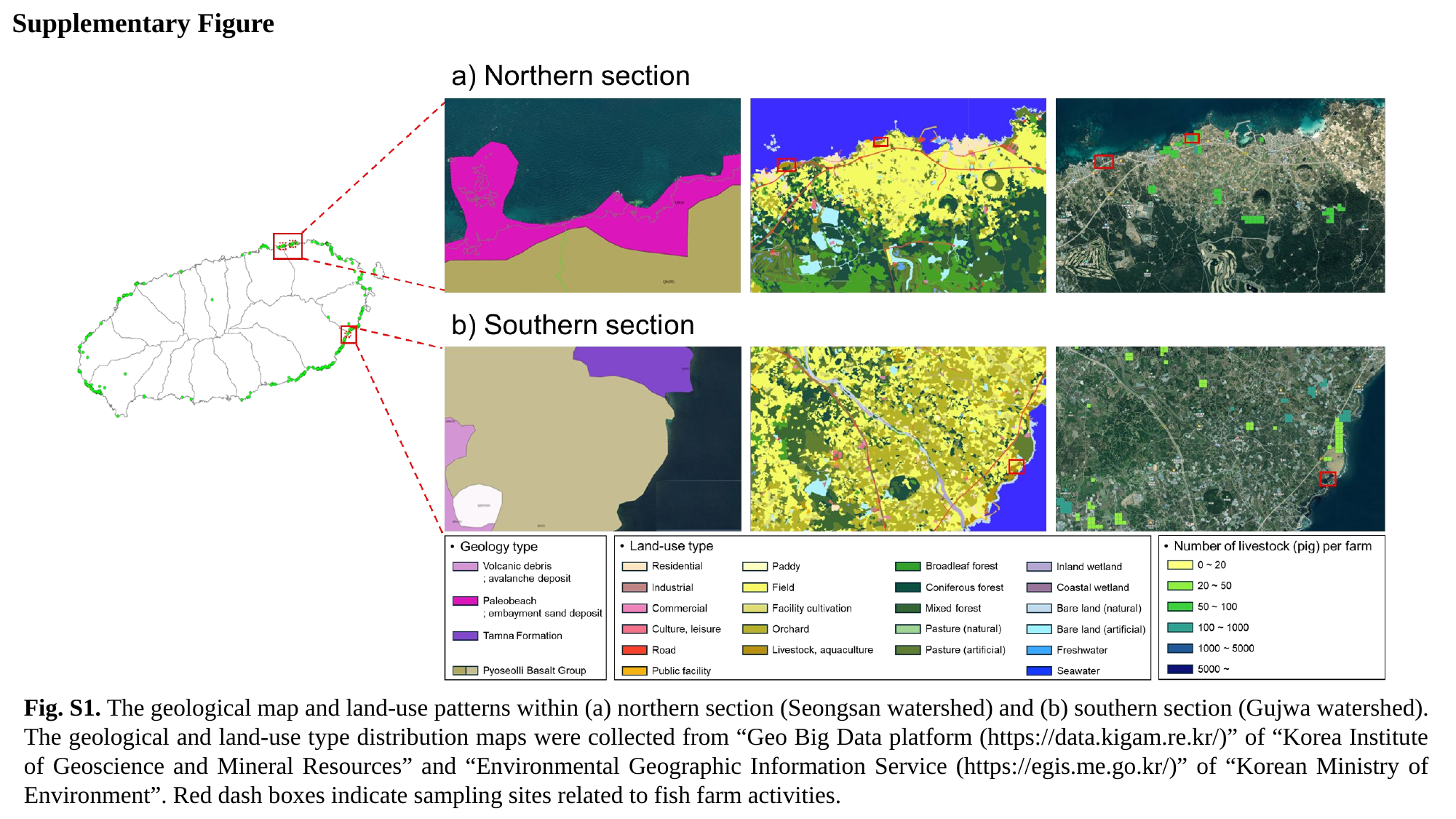

Supplementary Figure
Fig. S1. The geological map and land-use patterns within (a) northern section (Seongsan watershed) and (b) southern section (Gujwa watershed). The geological and land-use type distribution maps were collected from “Geo Big Data platform (https://data.kigam.re.kr/)” of “Korea Institute of Geoscience and Mineral Resources” and “Environmental Geographic Information Service (https://egis.me.go.kr/)” of “Korean Ministry of Environment”. Red dash boxes indicate sampling sites related to fish farm activities.

## Slide 2
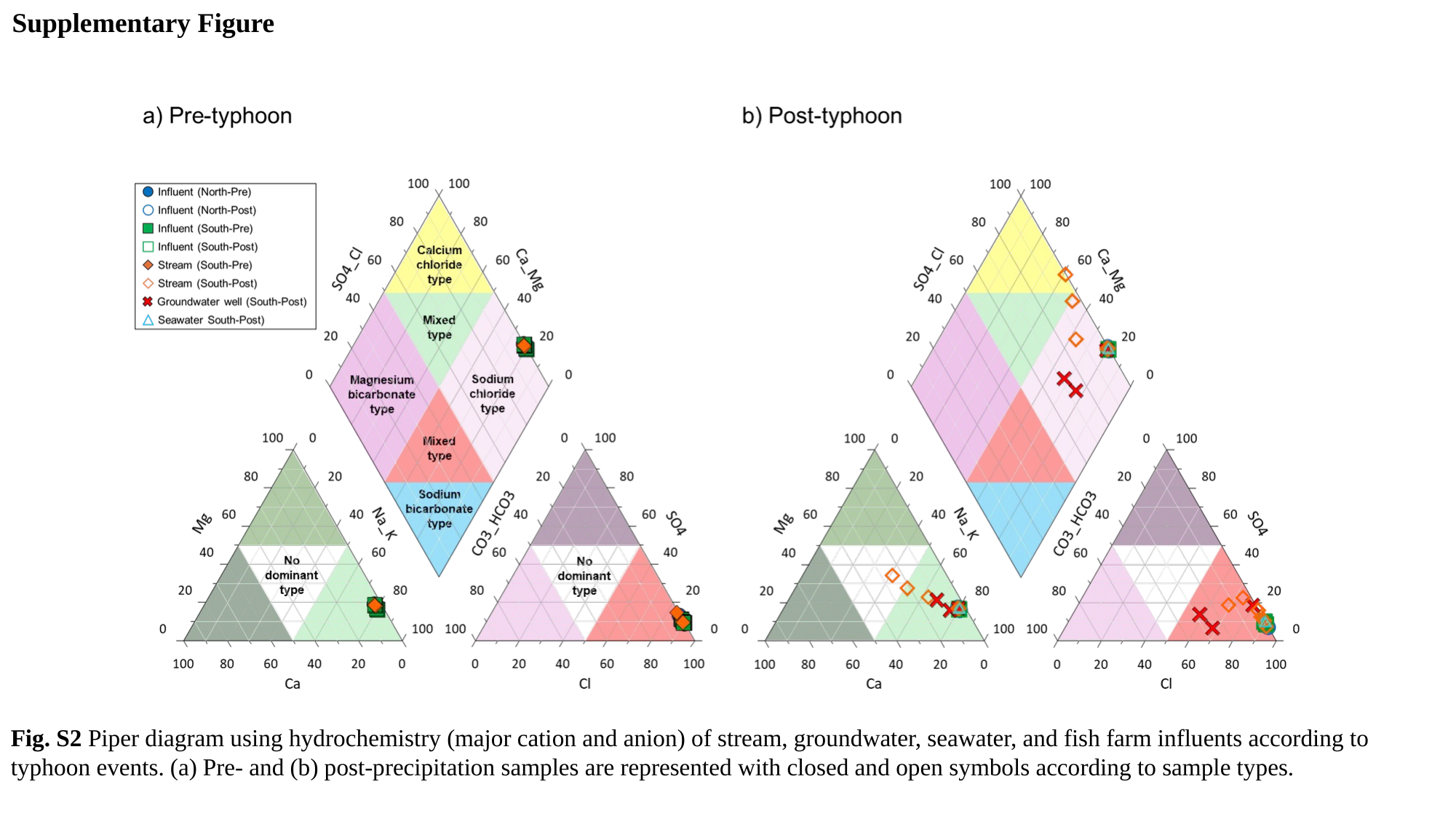

Supplementary Figure
Fig. S2 Piper diagram using hydrochemistry (major cation and anion) of stream, groundwater, seawater, and fish farm influents according to typhoon events. (a) Pre- and (b) post-precipitation samples are represented with closed and open symbols according to sample types.

## Slide 3
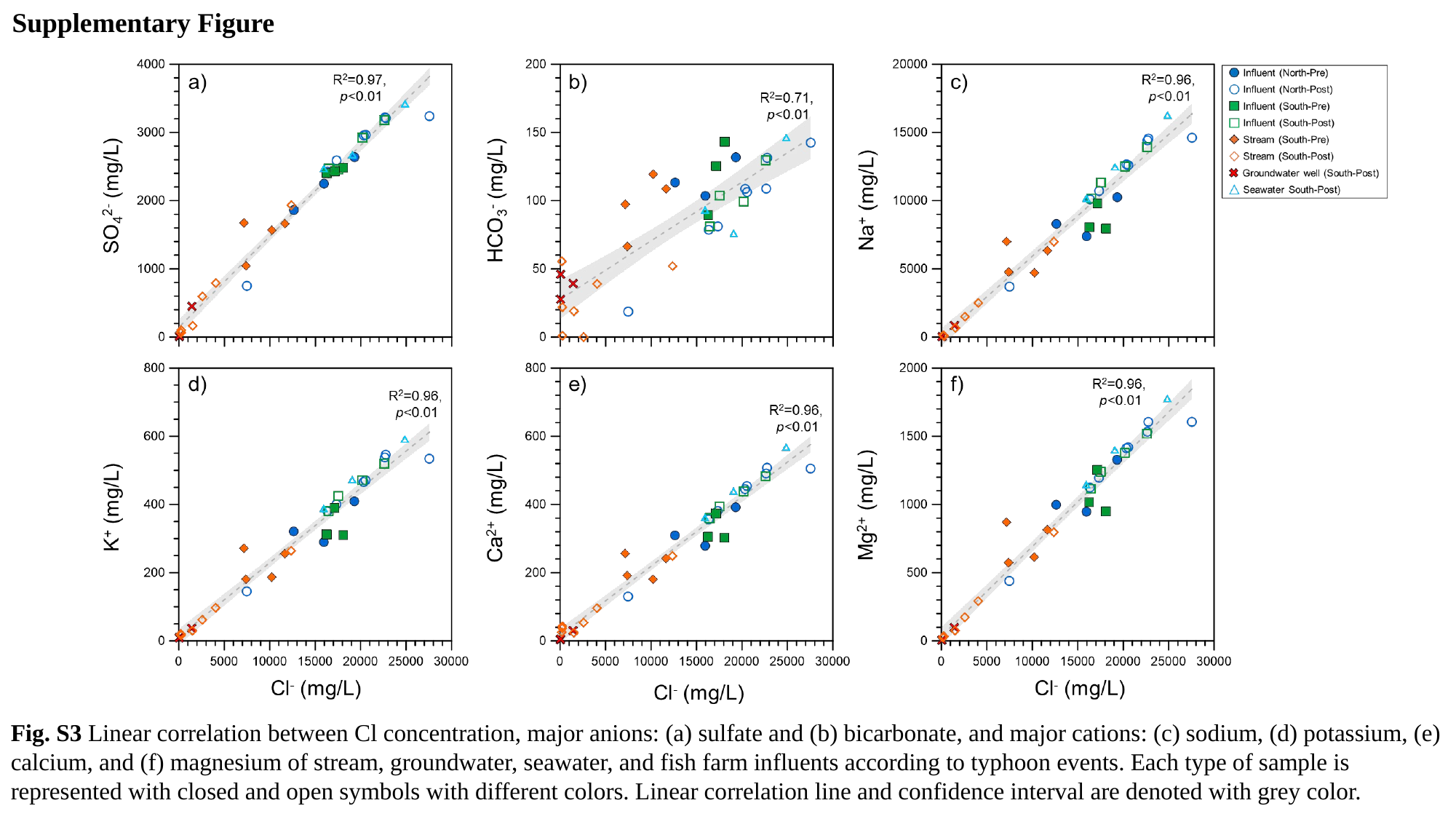

Supplementary Figure
Fig. S3 Linear correlation between Cl concentration, major anions: (a) sulfate and (b) bicarbonate, and major cations: (c) sodium, (d) potassium, (e) calcium, and (f) magnesium of stream, groundwater, seawater, and fish farm influents according to typhoon events. Each type of sample is represented with closed and open symbols with different colors. Linear correlation line and confidence interval are denoted with grey color.

## Slide 4
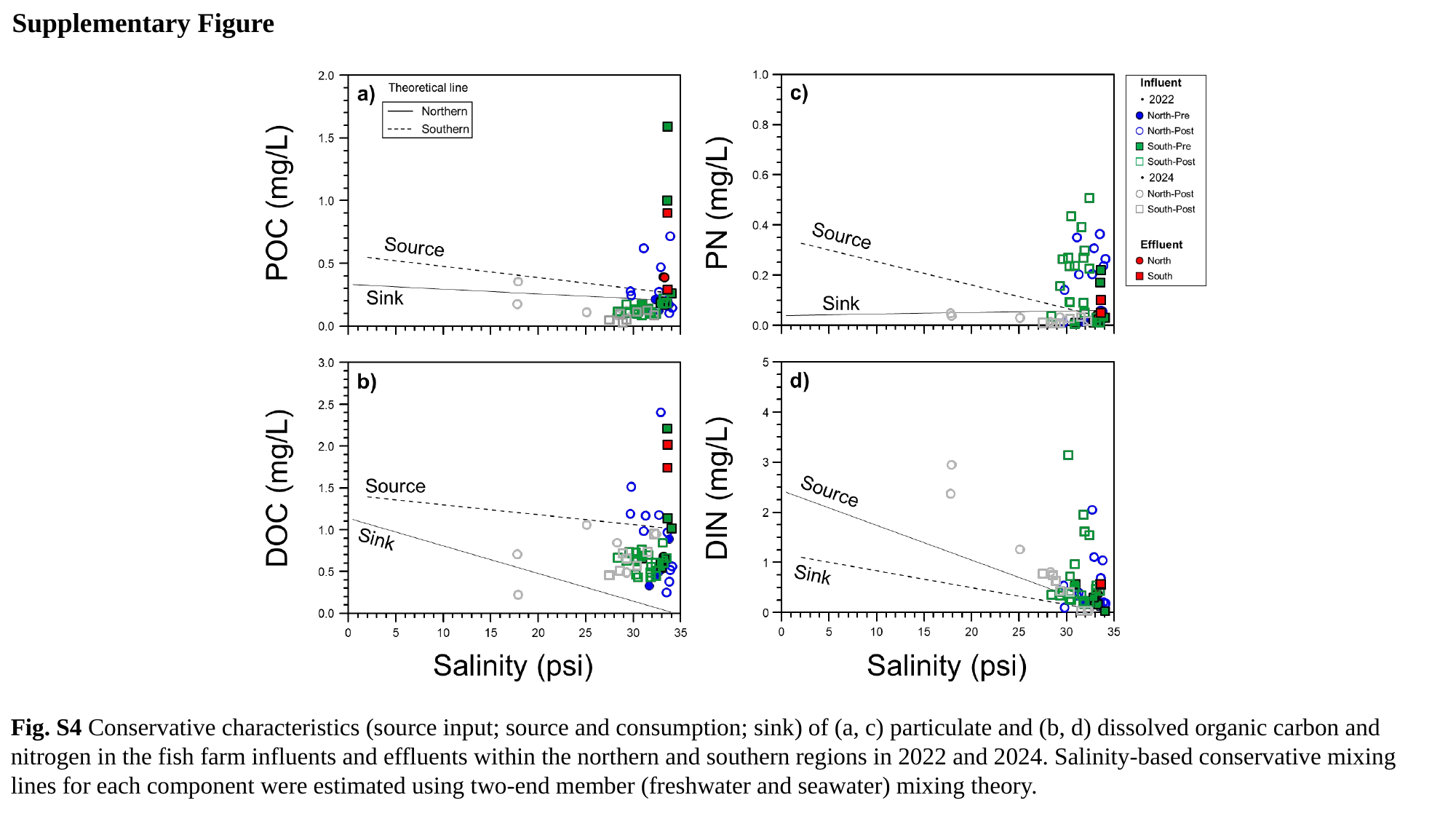

Supplementary Figure
Fig. S4 Conservative characteristics (source input; source and consumption; sink) of (a, c) particulate and (b, d) dissolved organic carbon and nitrogen in the fish farm influents and effluents within the northern and southern regions in 2022 and 2024. Salinity-based conservative mixing lines for each component were estimated using two-end member (freshwater and seawater) mixing theory.

## Slide 5
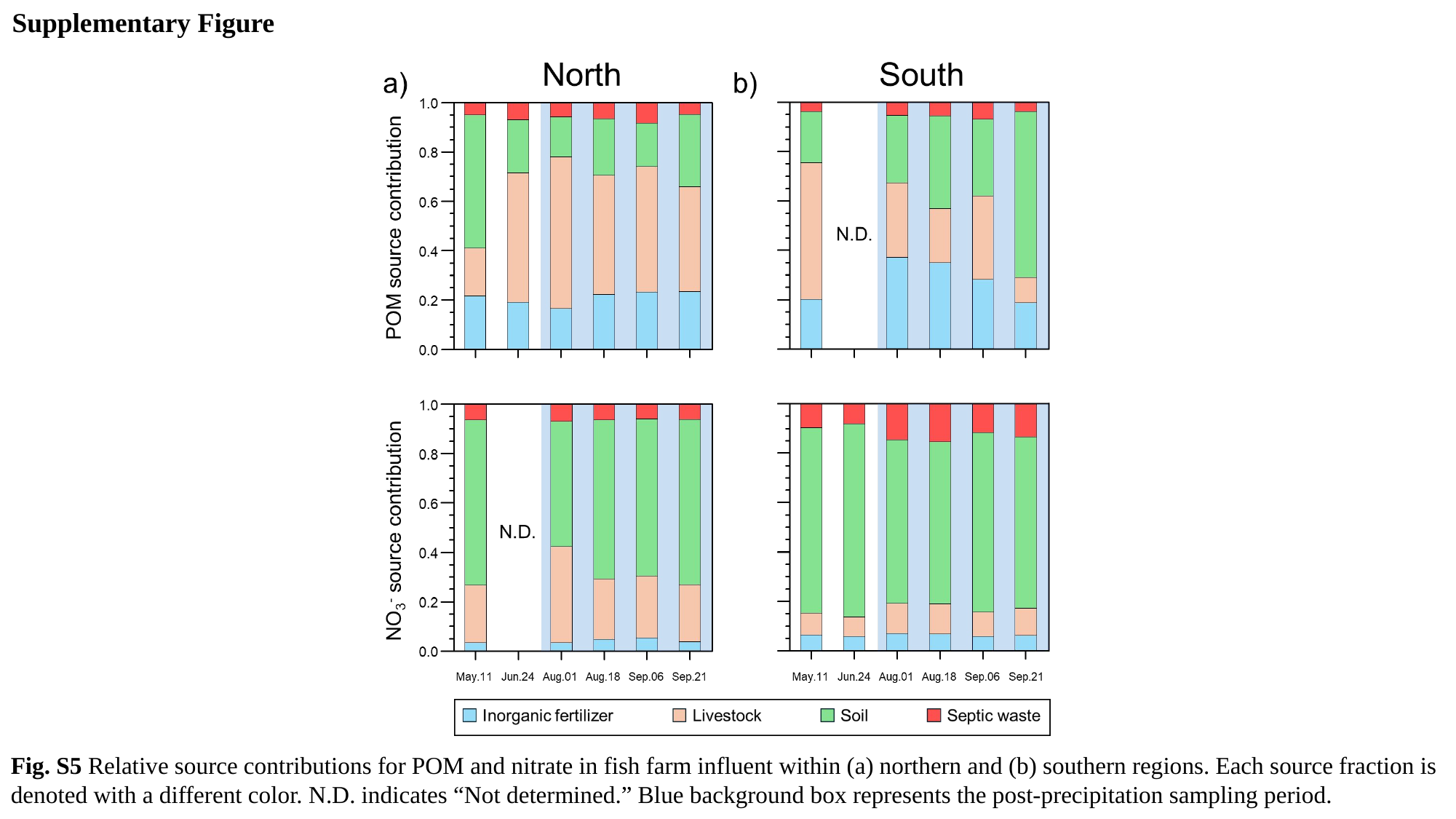

Supplementary Figure
Fig. S5 Relative source contributions for POM and nitrate in fish farm influent within (a) northern and (b) southern regions. Each source fraction is denoted with a different color. N.D. indicates “Not determined.” Blue background box represents the post-precipitation sampling period.
